# Supplementary material for: Implementation of the national Getting it Right First Time orthopaedic programme in England: a qualitative case study analysis
Source: BMJ Open. 2023 Feb 24;13(2):e066303. doi: 10.1136/bmjopen-2022-066303 (PMC9972449; doi:10.1136/bmjopen-2022-066303)
Supplement: Supplementary data [file bmjopen-2022-066303supp001.pdf]

Topic guide T1 and T2 – format amended for publication

GIRFT: T1 Final topic guide for provider interviews

Background checks / intro:

- Check respondent aware of aims of the study (e.g., reviewed information sheet)
- Seek permission to record.
- Confirm role in the Trust.

Priority questions are highlighted in yellow

A) Experience of GIRFT programme and site visits

1. Please describe your current understanding of the GIRFT programme in orthopaedics.
    - What do you think are the main problems or practices that the GIRFT team want to address? (N.B. establish knowledge of GIRFT priorities ***unprompted***)

**Intervention priority**	**Interviewer ticks all that are mentioned**
Procurement costs / Trust expenditure:<br>- Implants selection/supply (e.g. cemented / uncemented, prosthesis brand)<br>- Equipment costs (e.g. theatre loan kits)	
NHS Litigation costs	
Oxford hip / knee scores (OHS/OKS) which are indicative of primary total hip / knee replacements (PROMS adjusted health gains)	
5-year revisison rates	
Surgeon activity levels (i.e. % of surgeons undertaking less than 5 procedures per year)	
Readmission rates (i.e. for post surgical complications)	
Infection rates	
Length of Stay (primary total hip / knee replacements)	
Knee replacements following an arthroscopy after 1 year	
Other	[Enter details]

2. When did you first hear about GIRFT? [PROMPT:]
    - How was it introduced to you within this hospital? By whom?

3. Have you been involved in a GIRFT visit to this hospital? [**If yes – continue to Q.4**]:

4. Please describe your experience of this *first visit* by the GIRFT team ? [PROMPTS]:
    - When did this take place? [N.B. cross-check dates]
    - How long was the meeting?

Aspinal F, *et al. BMJ Open* 2023; 13:e066303. doi: 10.1136/bmjopen-2022-066303

Topic guide T1 and T2 – format amended for publication

- Did you prepare for the meeting in advance? If so, what did you do? How much time did you spend on this?
    i. Did you review the data pack sent in advance of the meeting? [N.B. cross-check dates]
- What was your impression of the data presented in the meeting? Did any aspects surprise you?
- Do you recall the main issues raised and discussed during that meeting?
- Was there anything pointed out by the GIRFT team that you found particularly helpful? And anything you found particularly challenging?
- What were your expectations before the visit? Did the meeting meet your expectations?
- Had you experienced anything like this before? If so, what was different about it?

5. What steps were taken following the *first* visit? [PROMPTS:]
    - Can you give us examples of any conversations that took place between senior managers and clinicians following the first GIRFT visit?
    - Who was involved in any decisions made after the visit?
    - What actions were agreed to take forward? (If any) [Fill out the table below]

Description of action taken	People involved	Time taken / duration	Costs involved / resources required for implementation (e.g. staff time, materials, capital)

6. Have you been involved in a GIRFT *re-visit* at this Trust? [PROMPTS:]
    - When did this take place? [N.B. cross-check dates]
    - How long was the meeting?
    - Did you prepare for the meeting in advance? If so, what did you do? How much time did you spend on this?
        i. Did you review the data sent in advance of the meeting? [N.B. cross-check dates]
    - Do you recall the main issues raised / discussed during this meeting?
    - Did it differ from the first meeting (if you were present)?
        - What steps were taken following this meeting? Who was involved in these decisions?

Aspinal F, *et al. BMJ Open* 2023; 13:e066303. doi: 10.1136/bmjopen-2022-066303

Topic guide T1 and T2 – format amended for publication

Description of action taken	People involved	Time taken / duration	Costs involved / resources required for implementation (e.g. staff time, materials, capital)

B) Other local quality improvement initiatives:

7. Are you involved in any national or local quality improvement initiatives focused on orthopaedic services? [PROMPTS – if yes:]
   - What are the aims of this initiative? [i.e. only non-GIRFT programmes]
   - When did the initiative begin?
   - What has been your level of involvement?
   - How is the initiative attempting to change orthopaedic services or practice?

C) The Model Hospital

8. Have you heard of the Model Hospital Orthopaedic dashboard? [PROMPTS:]
   - [If yes] What do you know about it?
   - Do you think it will help to makes changes in this Trust? How?

D) Changes processes and attribution to the GIRFT programme (i.e. changes to practice or processes, clinical and financial outcomes)

9. In your opinion, has GIRFT made a difference within this Trust? If so, please can you talk us through some examples? [PROMPTS]:
   - Have you made any changes to organisational practices around: 1) procurement; 2) litigation; 3) financial management; 4) clinical coding; 5) service delivery; 6) patient care?
   - What are the timescales for delivering these specific changes to practice? When did work begin? (i.e. explore short-term vs. medium-term vs. long-term)
   - Do you perceive any changes in how resources or finances are allocated in this Trust because of GIRFT interventions?
   - Have the GIRFT team helped you to deliver any of these changes?
   - Which changes in this hospital can be attributed to GIRFT in your opinion, and why?

Aspinal F, *et al. BMJ Open* 2023; 13:e066303. doi: 10.1136/bmjopen-2022-066303

Topic guide T1 and T2 – format amended for publication

- Can you tell us about any unintended consequences brought about as a result of GIRFT?
- Do you measure progress or success in relation to GIRFT within the Trust? [PROMPTS]:
- Do you measure cost savings arising from the GIRFT intervention?

E) Local context

10. Is there anything we should be aware of about the clinical coding system used at this Trust?
11. Is there anything that makes it difficult to implement the GIRFT recommendations in this site?
12. Is there anything - or anyone - that has really helped to support implementation of the GIRFT recommendations?

Wrap up & close

F) What improvements could be made to the GIRFT programme in future?

Topic guide T1 and T2 – format amended for publication

GIRFT: T2 Topic Guide for Follow-up Provider Interviews

Background checks / intro:

- Check respondent aware of aims of the study (e.g., reviewed information sheet)
- Seek permission to record.
- Confirm role in the Trust (especially if changed since T1 interview)
- [If possible, use presentation given at the GIRFT re-visit as a discussion prompt]

A) Implementation of GIRFT recommendations since last interview (T1)

1. Has this unit implemented any GIRFT recommendations in trauma and orthopaedics since [DATE OF T1 INTERVIEW]? Prompt list:

Changes in:	Tick those that apply
**Finance and procurement:**<br>• Procurement processes (e.g., new contracts, rationalisation of implants)<br>• Use of theatre loan kit (e.g., reduction in expenditure)	
**Infection control, bed management and theatre efficiency:**<br>• Ring fenced beds<br>• New infection control policies<br>• Data collection on infection rates<br>• Length of Stay (e.g., new protocols)<br>• Number of cases performed per day (e.g., minimum of 4 THR/TKR)	
**Rehabilitiation and physiotherapy:**<br>• Appointment of weekend cover<br>• New patient education materials	
**Surgeon-level clinical practice:**<br>• Surgeon minimum volumes (e.g. recommendations for thresholds for doing procedures)<br>• Cemented and / or uncemented implants in patients aged over 65/70<br>• Implant selection (e.g. bearings, type of supplier)	
**Team-or network-level practice:**<br>• Multi-disciplinary teams (e.g. for review of revisions within unit or regional network)<br>• Creation of committees / meetings (e.g. GIRFT implementation team)<br>• Review of performance or outcomes data to inform practice (e.g. NJR, litigation cases)	
**Workforce and service re-configuration:**<br>• Appointment of orthogeritrician/physiotherapists/shift in number of consultants (especially if low volume)<br>• 'Hot and cold' sites	

Topic guide T1 and T2 – format amended for publication

2. When did work [on X items listed above, if applicable] begin? What has facilitated this? [Explore *how* the work has been facilitated].

3. If you have not been able to implement any specific GIRFT recommendations, or decided not to, please explain why.

4. Have any GIRFT recommendations been particularly difficult to implement locally? If so, what factors have made implementation difficult? Why?

   Prompts:

• Winter bed pressures	Tick any that apply
• Workforce issues (e.g. lack of theatre staff/physiotherapists, recruitment and retention)	
• Lack of funds (e.g. for ring fenced beds)	
• Referral to treatment (RTT) targets	
• New tariff	
• Other	

5. Have there been any controversies locally around particular GIRFT recommendations in orthopaedics? (e.g., uncemented vs. cemented implant use) What impact have these had (if any)?

6. How are you measuring progress in relation to GIRFT within the Trust?

   PROMPTS:

   - Cost savings (e.g., theatre loan kit reduction)
   - NJR compliance
   - Less lower volume procedures
   - Lower infection rates

7. Can you tell us about any knock-on effects brought about as a result of GIRFT so far? (e.g., impact on trauma work in orthopaedics)

B) GIRFT orthopaedic re-visits 2018

1. To confirm, when did this site have a GIRFT orthopaedic re-visit with the national lead?

Topic guide T1 and T2 – format amended for publication

2. Who was invited to attend? Who was unable to attend? Did anyone else want to attend?

3. How did you prepare for the meeting? [Prompt]:
    - Preparation of presentation using GIRFT in advance of the visit

4. Did the most recent meeting differ from the first visit (if you were present)? If so, how?

5. Has anything happened since this re-visit? [Prompt]:
    - Are there GIRFT recommendations you are planning to implement in the next 6 months?

C) Other local quality improvement initiatives and research

1. Are you involved in any other quality improvement initiatives or research studies focused on orthopaedic services that relate to GIRFT orthopaedics?

D) The Model Hospital

1. Are you currently using the Model Hospital Orthopaedic dashboard? [PROMPTS:]
    - [If yes] What is your understanding?
    - Have you used it to make any changes in your Trust?
    - Do you think it will help to makes changes in this Trust? How?

E) GIRFT national and regional engagement

1. Have you liaised with any of the GIRFT implementation hubs / managers? [Prompt]:
    - What is your understanding of their role?
    - How this could this support implementation of GIRFT recommendations in your view?

F) Wrap up & close:

1. Are you aware of any changes in the national GIRFT programme since we last spoke?
2. PROMPT for awareness of evolution of national programme in relation to orthopaedics
3. Do you have any recommendations for improvements which could be made to the GIRFT programme?
4. Do you have any further comments?

Thanks and close

Aspinal F, *et al. BMJ Open* 2023; 13:e066303. doi: 10.1136/bmjopen-2022-066303
