# Supplementary material for: Implementation of the national Getting it Right First Time orthopaedic programme in England: a qualitative case study analysis
Source: BMJ Open. 2023 Feb 24;13(2):e066303. doi: 10.1136/bmjopen-2022-066303 (PMC9972449; doi:10.1136/bmjopen-2022-066303)
Supplement: Author's manuscript [file bmjopen-2022-066303.draft_revisions.pdf]

BMJ Open is committed to open peer review. As part of this commitment we make the peer review history of every article we publish publicly available.

When an article is published we post the peer reviewers' comments and the authors' responses online. We also post the versions of the paper that were used during peer review. These are the versions that the peer review comments apply to.

The versions of the paper that follow are the versions that were submitted during the peer review process. They are not the versions of record or the final published versions. They should not be cited or distributed as the published version of this manuscript.

BMJ Open is an open access journal and the full, final, typeset and author-corrected version of record of the manuscript is available on our site with no access controls, subscription charges or pay-per-view fees (http://bmjopen.bmj.com).

If you have any questions on BMJ Open's open peer review process please email info.bmjopen@bmj.com

BMJ Open

Implementation of the national Getting it Right First Time orthopaedic programme in England: a qualitative case study analysis.

Journal:	*BMJ Open*
Manuscript ID	bmjopen-2022-066303
Article Type:	Original research
Date Submitted by the Author:	04-Jul-2022

Complete List of Authors:	Aspinal, Fiona; University College London, Department of Applied Health Research<br>Ledger, Jean; University College London, Department of Applied Health Research<br>Jasim, Sarah; The London School of Economics and Political Science, Care Policy and Evaluation Centre<br>Mehta, Raj; University College London, NIHR ARC North Thames Research Advisory Panel, Department of Applied Health Research<br>Raine, Rosalind; University College London, Department of Applied Health Research<br>Fulop, Naomi; University College London, Department of Applied Health Research<br>Barratt, Helen; University College London, Department of Applied Health Research

Keywords:	Quality in health care < HEALTH SERVICES ADMINISTRATION & MANAGEMENT, Adult orthopaedics < ORTHOPAEDIC & TRAUMA SURGERY, QUALITATIVE RESEARCH, Orthopaedic & trauma surgery < SURGERY

SCHOLARONE™
Manuscripts

BMJ

I, the Submitting Author has the right to grant and does grant on behalf of all authors of the Work (as defined in the below author licence), an exclusive licence and/or a non-exclusive licence for contributions from authors who are: i) UK Crown employees; ii) where BMJ has agreed a CC-BY licence shall apply, and/or iii) in accordance with the terms applicable for US Federal Government officers or employees acting as part of their official duties; on a worldwide, perpetual, irrevocable, royalty-free basis to BMJ Publishing Group Ltd ("BMJ") its licensees and where the relevant Journal is co-owned by BMJ to the co-owners of the Journal, to publish the Work in this journal and any other BMJ products and to exploit all rights, as set out in our licence.

The Submitting Author accepts and understands that any supply made under these terms is made by BMJ to the Submitting Author unless you are acting as an employee on behalf of your employer or a postgraduate student of an affiliated institution which is paying any applicable article publishing charge ("APC") for Open Access articles. Where the Submitting Author wishes to make the Work available on an Open Access basis (and intends to pay the relevant APC), the terms of reuse of such Open Access shall be governed by a Creative Commons licence – details of these licences and which Creative Commons licence will apply to this Work are set out in our licence referred to above.

Other than as permitted in any relevant BMJ Author's Self Archiving Policies, I confirm this Work has not been accepted for publication elsewhere, is not being considered for publication elsewhere and does not duplicate material already published. I confirm all authors consent to publication of this Work and authorise the granting of this licence.

For peer review only

Implementation of the national Getting it Right First Time orthopaedic programme in England: a qualitative case study analysis.

*Fiona Aspinal, Department of Applied Health Research, University College London, 1-19 Torrington Place, London, WC1E 7HB, UK f.aspinal@ucl.ac.uk

Jean Ledger, Department of Applied Health Research, University College London, UK

Sarah Jasim, Care Policy and Evaluation Centre (CPEC), London School of Economics and Political Science, London, UK

Raj Mehta, Patient Advisor, NIHR ARC North Thames Research Advisory Panel, Department of Applied Health Research, University College London, UK

Rosalind Raine, Department of Applied Health Research, University College London, London, UK

Naomi Fulop, Department of Applied Health Research, University College London, London, UK

Helen Barratt, Department of Applied Health Research, University College London, London, UK

*corresponding author

Word count: 4554 (406 are quotes)

For peer review only

For peer review only - http://bmjopen.bmj.com/site/about/guidelines.xhtml

ABSTRACT

Objective: To describe the implementation and impact of the Getting it Right First Time (GIRFT) national orthopaedic improvement programme at the level of individual National Health Service (NHS) Trusts.

Design: Qualitative case studies conducted at six NHS Trusts, as part of a mixed methods evaluation of GIRFT.

Setting: NHS elective orthopaedic surgery in England.

Participants: 59 NHS staff

Intervention: Improvement bundle, including bespoke routine performance data and improvement recommendations for each organisation, delivered via 'deep-dive' visits to NHS Trusts by a senior orthopaedic clinician.

Results: Although all case study sites had made improvements to care, very few of these were reportedly a direct consequence of GIRFT. A range of factors, operating at three different levels, influenced their ability to implement GIRFT recommendations: at the level of the orthopaedic team (micro - e.g. how individuals perceived the intervention); the wider Trust (meso - e.g. competition for theatre/bed space); and the health economy more broadly (macro - e.g. requirements to form local networks). Some sites used GIRFT evidence to support arguments for change which helped cement and formalise existing plans. However, where GIRFT measures were not a Trust priority because of more immediate demands– for example, financial and bed pressures – it was less likely to influence change.

Conclusion: Dynamic relationships between the different contextual factors, within and between the three levels, can impact the effectiveness of a large-scale improvement intervention and may account for variations in implementation outcomes in different settings. When designing an intervention, those leading future improvement programmes should consider how it sits in relation to these three contextual levels and the interactions that may occur between them.

For peer review only - http://bmjopen.bmj.com/site/about/guidelines.xhtml

Strengths and limitations of this study

- This work is part of the first independent evaluation of the high profile, national improvement programme, Getting it Right First Time (GIRFT).
- Observing 'deep-dive' visits to Trusts by the GIRFT team, reviewing local documentation, and interviewing clinicians and managers, enabled us to explore in-depth the factors that influenced the implementation and impact of GIRFT at a local level.
- Although we only studied the process of implementation at six NHS provider Trusts, our case study approach created a rich and detailed picture of changes over time, in a variety of settings.
- We spoke to a range of clinicians and managers at each site, but it is possible that we may not have captured all the improvements at each site, not least because the interviews took place several months after the first GIRFT visits creating a risk of recall bias.

For peer review only

For peer review only - http://bmjopen.bmj.com/site/about/guidelines.xhtml

BACKGROUND

Orthopaedic procedures such as total hip and total knee replacement are among the commonest surgical procedures in the UK[1] and worldwide.[2,3] Demand is likely to increase given ageing populations and the associated increased need for arthroplasty surgery. This is likely to have a significant impact on health systems globally[4] including the National Health Service (NHS) in England, which already faces a backlog of planned surgery due to the COVID-19 pandemic.[5]

'Getting it Right First Time' (GIRFT) is one of the largest improvement programmes in the NHS. It began in orthopaedics in 2012.[6] Following government investment of over £62m, it now operates in 44 different specialties or clinical workstreams across England.[7] GIRFT aims to draw clinicians and managers together to reduce unwarranted variation, improve outcomes and maximise efficiency.[7] This includes changing practice (e.g. prosthesis choice); organisational processes (e.g. procurement, length of stay); and service delivery (e.g. ring-fenced beds).[8]Clinical leadership is fundamental: GIRFT was established by a senior surgeon, who leads the orthopaedic workstream and chairs the wider programme. Each workstream is chaired by a clinical lead from the relevant specialty. The programme includes components operating locally (i.e. at NHS provider Trusts) and nationally (i.e. across England). Local components include 'deep dive' visits to Trusts, while national components include reports describing how variations can be addressed. Before each visit, Trusts are sent a 'datapack,' collated by GIRFT, describing their performance on over 100 variables, drawn from sources including Hospital Episode Statistics and the National Joint Registry. Datapacks cover use of evidence-based procedures and costs, and facilitate comparison to national and peer group averages. Discussion at the visits is driven by the datapack and tailored to the Trust. Attendees, comprising clinicians, managers and other relevant professionals, identify where improvements could be made. Although nationally there have been improvements in orthopaedic care over the last decade, these began before GIRFT. Other concurrent initiatives also targeted similar outcomes (e.g. Lord Carter's review of NHS efficiency and NHS Right Care), so it is not possible to quantify the impact of GIRFT itself. Estimations of the additional impact of GIRFT deep dive Trusts visits, found a mix of positive and negative effects that were generally small compared with overall improvement trends.[9]

It is increasingly recognised that, at an organisational level (e.g. within individual NHS Trusts), the impact of an intervention such as GIRFT, is shaped by multiple, contextual factors.[10] Context is defined as the underlying systems, culture and circumstances of the environment in which an intervention is implemented.[11] Contextual factors operate at three levels: micro (individual); meso (organisational); and macro-level (external/policy). At the micro-level of individuals in healthcare settings trying to implement change, collaboration must be fostered to facilitate change[12] and clinical leaders are often able to exert particular influence because of their professional credibility.[13] At the meso-level, health care organisations themselves play a central role in improvement.[14] Relevant factors may include processes (e.g. how strategy is developed); leadership (e.g. how leaders engage with their external context), and underlying features (e.g. leadership stability), all of which vary between different settings.[10] Outer 'macro-level' context refers to influences beyond the organisation, such as government policy and regulation, which can have a strong influence on how organisations approach improvement.[15] Whilst the intervention and local context interact to determine whether and how improvement occurs, it is increasingly

For peer review only - http://bmjopen.bmj.com/site/about/guidelines.xhtml

recognised that interactions between the three levels of context can also serve to effect improvement or confound it.[10]

Although the influence of context on improvement is widely acknowledged, understanding of its role is still limited.[14] In this paper, we describe the implementation and impact of GIRFT at the level of individual Trusts. We explore both the contextual factors that influenced implementation and the ways in which the interaction of factors operating at different levels determined whether and how change took place. In doing so, we contribute to knowledge about how context influences how national improvement programmes effect change, and to inform the design and development of context-sensitive interventions in the future. This research was part of a wider mixed methods evaluation of the GIRFT orthopaedic programme.[9,16]

DESIGN

We used a case study approach to examine the different ways Trusts implemented GIRFT and how this influenced the impact of the programme locally. Case studies allow complex phenomena such as improvement to be studied in-depth, including the role of context in this process. Undertaking multiple case studies can increase the transferability of data to other situations and contexts.[17,18]

Case selection

We identified six case study sites, all NHS Trusts participating in the GIRFT orthopaedic programme. We selected sites purposively to represent a spread of hospital types (district general and teaching hospitals); different levels of performance on GIRFT metrics and engagement with the GIRFT programme at the time of recruitment; and settings (region of England; rural versus urban).

In July 2016, the GIRFT programme team provided a list of Trusts grouped according to their own qualitative assessment of a) performance on GIRFT metrics at that time and b) extent to which they perceived managers and clinicians were committed to implementing change together. From this, we identified an initial list of six potential sites to meet the wider criteria and emailed the clinical lead for trauma and orthopaedics at each. Four responded positively, but we were unable to secure engagement at the other two. We returned to the list of potential sites to identify, and then recruit, two further Trusts.

Our collaboration with the GIRFT team to identify the level of engagement of Trusts with the GIRFT programme enabled us to draw on their knowledge of orthopaedic performance and key individuals at Trusts. However, the final selection of sites (and all other research decisions) was made independently. This independence was made clear to participating sites. Data collection was only carried out by members of the research team. The GIRFT team were not informed which sites took part, and all sites were anonymised throughout the study.

METHODS

Case study research draws on a variety of data sources to construct a detailed picture of phenomena (in this case, implementation and impact of the GIRFT programme) in their ‘natural setting’.[19] In each of the six sites, data were collected via non-participant observation of GIRFT visits, semi-structured telephone and in-person interviews, and collection and analysis of local literature/documents. Data were collected between Oct 2016 and May 2019.

For peer review only - http://bmjopen.bmj.com/site/about/guidelines.xhtml

Interviews

We conducted a round of semi-structured interviews with orthopaedic surgeons, managers and other key staff who were present at the first Trust visit and/or knowledgeable about improvement work locally. The interviews covered: (i) organisational engagement with the GIRFT programme and the reasons for this; (ii) practice and service delivery changes, particularly those concerning key GIRFT metrics such as ring-fenced beds, low volume surgeons, fixation methods and procurement; and (iii) perceptions of the impact of GIRFT on any changes. The topic guide was piloted prior to the interviews and refined iteratively as the study progressed to take account of emerging findings. A second round of interviews was conducted following the Trusts' second deep-dive visit. Similar topics were covered, with a focus on change over time including implementation of GIRFT recommendations, and continued engagement with the programme. Some people were interviewed at both time points and some at a single time point. All interviewees gave informed consent. Interviews lasted between 20 minutes and 1 hour and were digitally recorded for professional transcription in full.

Observation of deep-dive visits

Researchers (JL & SJ) attended and observed all GIRFT orthopaedic visits that took place at case study sites during the data collection period. This provided an understanding of the data being presented to the sites by the GIRFT team; the local context; and local receptiveness/engagement with the GIRFT programme. Researchers kept field journals for recording notes about attendee behaviours and interactions during meeting observations. They also attended deep-dive visits at non-case study Trusts and GIRFT events to better understand the wider programme and its delivery. This helped obscure the identities of participating sites from the GIRFT team.

Local documents

We collated documents from each site, including GIRFT data-packs and presentations, Trust annual reports, and local documents about, for example, other improvement programmes. These provided information about local approaches to improvement; competing/complementary priorities; the local population; Trust services and performance; and wider strategic decisions.

Patient and public involvement

Patient and public advice on the design and conduct of this research was provided via the National Institute for Health and Care research (NIHR) Applied Research Collaboration (ARC) North Thames' Research Advisory Panel. A patient representative (RM) was a core member of the research advisory group throughout the project.

DATA ANALYSIS

Interviews were subjected to thematic analysis.[20] Data from documents and observations were reviewed to help interpret and contextualise interview data. Observation notes were included in thematic analysis where appropriate.

The initial coding frame combined inductive and deductive approaches. It focussed on whether and how sites engaged with GIRFT; reported 'improvements' in care; and the factors affecting this. Cross-

For peer review only - http://bmjopen.bmj.com/site/about/guidelines.xhtml

case comparison drew on the interaction between different levels of context (micro, meso and macro) to help explain how Trusts responded to a large-scale quality improvement programme.

Quotations used in the findings are illustrative of the points being made and representative of the wider patterns in the data.[21]

FINDINGS

Information about participating sites, interviewees and data sources are presented before the findings from the case study analysis.

The six case sites represent a spread of Trust types, rurality, level of engagement with the programme and geographical region. To protect the anonymity of sites, we have summarised their characteristics data in Table 1.

Table 1: Participating case sites

Criteria	Category	Counts
**Type of Trust**	District General	3
	Teaching	3
**Setting**[1]	Rural	2
	Urban	3
	Mixed	1
**Level of engagement with the GIRFT programme at time of recruitment**	High	2
	Medium	2
	Low	2
**Region of England**	East of England	1
	London	1
	North East & Yorkshire	1
	South East	2
	South West	1

[1]defined by interviewees and/or local public health annual reports.

We interviewed 59 key stakeholders across the six Trusts, including orthopaedic surgeons (some of whom were also managers), other clinical staff, and hospital managers (some of whom retained a clinical role), including at least one representatives of Trusts' executive leadership team in each site. Those interviewees with a joint clinical and managerial role, are referred to here as a 'hybrid manager'. Forty-six people were interviewed at Time 1 and 13 at time two. Some people preferred to be interviewed together with one or more colleagues: in Site 1, one interview was conducted with two people and another with three, and in Site 2 one of the interviews included two people. Table 2 shows the interviewees at each time point.

For peer review only - http://bmjopen.bmj.com/site/about/guidelines.xhtml

Table 2: Interviewees per case site

	Time	Surgeon	Nurse	Hybrid*	Manager	Commissioner	Total per time point	Total per site
**Site1**	T1	1		5	2	3	11	**14**
	T2	1		1		1	3	
**Site2**	T1	2		1	5		8	**11**
	T2	1			2		3	
**Site3**	T1	3	1	1			5	**7**
	T2			1	1		2	
**Site4**	T1	2		3	2		7	**9**
	T2	2					2	
**Site5**	T1	6			1		7	**8**
	T2				1		1	
**Site6**	T1	2		2	4		8	**10**
	T2				2		2	
**Total**		**20**	**1**	**14**	**20**	**4**	**T1 = 46**<br>**T2 = 13**	**59**

*Hybrid includes clinician/managers

Case study analysis

Before each 'deep dive' visit, Trusts were sent a 'datapack' describing their performance on over 100 variables. Each visit was led by the GIRFT orthopaedic clinical lead. Discussion was driven by the datapack, so recommendations were specific to each Trust. Here, we explore first the factors that affected the adoption and implementation of GIRFT, including micro, meso and macro-level contextual influences. We then describe changes made to orthopaedic care at case study sites and the extent to which these were attributable to GIRFT.

Micro-level contextual factors

At the individual level, managers and clinicians who were interviewed demonstrated good understanding of GIRFT and its aims. They described a variety of routes through which they first heard about the programme, including via the British Orthopaedic Association (BOA); colleagues; the initial GIRFT report;[6] and attending deep-dive visits at their Trust. However, many claimed that neither the underlying concepts nor delivery approach were novel:

> *Well, I suppose I became aware of the concepts that underpin GIRFT before GIRFT existed.* (Hybrid professional, Site 6)

Overall, interviewees across all sites supported what the programme sought to do – reducing unwarranted variation, maximising quality and promoting efficiency – and this made them more open to adopting the programme. As one participant said:

> *Personally, I thought "At last!" It is fantastic.* (Hybrid professional, Site 3)

For peer review only - http://bmjopen.bmj.com/site/about/guidelines.xhtml

Two major factors though influenced how individuals perceived the intervention itself. The first was how they viewed the programme as a whole. At three sites, interviewees argued that their deep-dive visits focussed on costs rather than quality. In one, for example, a senior manager argued that while the Trust always emphasised quality over cost, their deep-dive visit focussed on:

> *…money and efficiency and how many patients you can get through and how cheap you can buy prostheses for that work…* (Hybrid manager, Site 4).

In the sites where GIRFT was perceived to focus on costs, there was low engagement, especially from surgeons, although in one site (Site 6) this perceived cost focus triggered high engagement from managers.

The second factor was the approach taken by the GIRFT clinical lead. While almost all interviewees acknowledged his expertise, some described how his particular approach in deep-dive visits affected their opinion of, and willingness to engage with, the programme. Some admired his style for '*not tak[ing] any of [the surgeons'] rubbish*' (Manager, Site 2), others found him '*supportive'* (Surgeon, Site 5) and '*very compelling*' (Hybrid manager, Site 6). However, others considered him '*adversarial'* (Manager, Site 6) and '*patronising'* (Manager, Site 4).

Those interviewees aligned with the latter views expressed more reluctance to actively engage with GIRFT. Where these individuals had power to facilitate or resist engagement with the programme, these views negatively impacted adoption and implementation locally.

Equally, interviewees from all six sites argued that their visits had not taken the form of a two-way dialogue, limiting opportunities for attendees to question and challenge data, approaches and metrics. Consequently, at least some disengaged with the process, risking delivery of the programme. As one manager noted:

> *I'm sure [the surgeons] would all value [the GIRFT] process and the information, but it has to be delivered in a way that's open to discussion and negotiation and mutual understanding.* (Manager, Site 4).

GIRFT is now being rolled out to over 40 other specialities, each of which has separate clinical lead. Interviewees who had attended visits in other specialities described different delivery approaches, which enabled two-way dialogue and a more flexible approach. This, they suggested, helped maintain their engagement.

The need for flexibility in implementing GIRFT recommendations was also highlighted. For example, interviewees felt that there was an expectation that Trusts would change practice simply to comply with GIRFT priorities, even where their outcomes were already above average and there was no cost benefit to derive from the change. For example, at Sites 1, 2, 3 and 5, at least some surgeons were reluctant to switch to cemented fixation for all hip arthroplasty patients over 65 because it did not allow for surgeon responsiveness (site 5) or consider the financial or wider organisational consequences of the additional training that would be required for staff (site 1, 2, 3, 5).

For peer review only - http://bmjopen.bmj.com/site/about/guidelines.xhtml

The BOA's guidance[22] about implementing GIRFT recognised the need for some flexibility, and was welcomed by interviewees:

> *Everybody in orthopaedics thinks that GIRFT is a good idea, but not everybody agrees with everything [the programme lead] says, so the [BOA] implementation document was a way of making [it] palatable to the wider profession*. (Hybrid manager, Site 3).

Meso-level contextual factors

At an organisational level, four of the sites had a long-embedded culture of quality improvement, including engaging with external quality initiatives in orthopaedics and across the Trust more widely. These Trusts already had internal processes for analysing performance data to inform service delivery and practice:

> *…the staff are very pro-department and the Trust and they want to make improvements and make the best journey and the best outcomes for our patients. So they do come up with ideas of how we can improve. The consultants are also very proactive with regards to what improvements can be made and how the team can be best set up to get the best outcomes for the patients.* (Lead nurse, Site 1)

In several of the case sites, Trust managers commented that engagement in the GIRFT programme was useful because it provided a '*free, independent quality audit*' (e.g. Manager, Site 4) and '*benchmarking*' (e.g. Surgeon, Site 4). More importantly GIRFT analysed more local data than some had capacity or resources to analyse internally. Already being open to instigating evidence-informed change did not always lead to GIRFT-instigated change, but it could catalyse change processes so that results were experienced more quickly. For example, in case site 3, GIRFT was used to expedite existing plans to streamline implant selection.

Nevertheless, across the sites, there was a feeling that GIRFT recommendations did not acknowledge the impact that Trust context could have on change. This included how the physical structure of a Trust could act as a barrier to improvement, even when individuals were convinced change was needed. For example, some interviewees argued that it was easier for multi-site orthopaedic services to separate trauma and elective care and, in turn, to ring-fence beds. In contrast, single-site Trusts often struggled to set aside beds for elective orthopaedics, especially during winter months:

> *So, like ring-fenced elective beds, that's an absolute critical requirement and recommendation of the GIRFT project. [Programme lead] talks about it a lot. And we all agree with that, but we still are unable to ring-fence elective orthopaedic beds [b]ecause we are a massive hospital, an acute Trust, and lots of people are dying of flu and so on and so on. Those beds are needed to house the dying* [elderly] *in the winter.* (Surgeon, Site 5)

For peer review only - http://bmjopen.bmj.com/site/about/guidelines.xhtml

GIRFT recommendations linked to theatre capacity and bed usage, such as increasing the number of orthopaedic procedures per session or repatriating surgery from Any Qualified Providers (AQP), also encountered barriers. However, these were more about organisational complexity than organisational configuration. For example, interviewees talked about needing to *'vy[e] with urology, colorectal, vascular and all the other specialities'* (Hybrid manager, Site 6) to access theatre time, as well as negotiate bed availability across wards and arrange appropriate staffing.

Interviewees also noted that the complex planning needed to facilitate improvement in a busy acute Trust, means that change takes time. For example, interviewees in Site 6 described it taking eight months for ring-fenced beds to be implemented after Trust management had agreed because of other Trust priorities, space and bed demand-management, and associated cross-speciality negotiations (Surgeon, Site 6).

GIRFT's national peer benchmarking could, however, *'raise awareness'* (Surgeon, Site 2) and *'leverage support'* (Surgeon, Site 4) at a high level within Trusts that then catalyse improvement. As witnessed in Site 6, GIRFT benchmarking data prompted the site to institute ring-fenced beds, something that had been requested by clinicians, but had been on the backburner at the Trust for some years.

Macro-level contextual factors

Implementation of GIRFT recommendations by Trusts was also impacted by factors outside the organisation. For example, establishing networks to manage complex and low-volume procedures could be hampered by complex dynamics at a regional level. This was especially the case where CCG boundary and hospital catchment areas and/or the regional Sustainability and Transformation Partnership's (STP – now reorganised as Integrated Care Systems (ICS)) footprint were not aligned. In one site, GIRFT feedback was instrumental in a network being initiated, but this quickly broke down due to changing regional structures.

> [After the deep-dive visit] *we straight away looked at forming networks amongst the hospitals [to organise] the low volume procedures. Unfortunately, it's broken down… So, that's why, politically, it broke down, because we had to align with our own STPs. So, politically speaking, we had to withdraw from that and try and start up a new alliance with* [other Trusts] *in region*. (Surgeon, Site 2)

Impact

Interviewees were asked to describe any changes to orthopaedic care in recent years. Table 3 lists the changes that had been made in recent years and shows whether interviewees attributed this to GIRFT. Only two of the sites (Sites 1 & 2) confirmed that changes that had occurred were a direct result of GIRFT. Indeed, many indicated that their Trusts were working towards or had already addressed some of the GIRFT priorities before the programme started in 2012. This had occurred in response to organisational or regional performance management processes (Sites 1-6), organisational changes (e.g. leadership changes, Sustainability and Transformation Partnership (STP) requirements or CQC status) (Sites 1, 3 & 6) and/or preparing or delivering musculoskeletal pathway bids/contracts (Sites 1, 2, & 6). Thus, while many changes aligned with GIRFT, few were actually attributable to the programme directly (see Table 3).

For peer review only - http://bmjopen.bmj.com/site/about/guidelines.xhtml

Table 3: Changes reported by participants in sites and their attribution to GIRFT

	Recent change attributed to GIRFT by study participants			
**Sites**	**Fully**	**Partly**	**Possibly**	**Not**
**1**	Fixation method<br>Network development			
**2**	Fixation method<br>Network development			Procurement rationalisation<br>Arthroscopy reduction
**3**			Fixation method<br>Network development<br>Implant rationalisation	Length of Stay
**4**			Procurement rationalisation	Fixation method<br>Low-volume surgeons<br>Network development<br>Infection rates
**5**			Ring-fenced beds	Procurement rationalisation<br>Low-volume surgeons<br>Network development
**6**		Ring-fenced beds<br>Waiting list reduction		Low-volume surgeons<br>Network development

For peer review only

However, where change was already planned or in progress, some participants found the GIRFT programme and deep-dive visits useful for cementing and formalising plans (for example, formalising the existing regional MDT network in Site 5), catalysing action (network development in Site 1), and leveraging support for change (for example, surgeons leveraging management support for ring-fenced beds in Site 6).

In four of the sites, there was a question mark over the influence of GIRFT because interviewees were unsure of the relative contribution of the programme when other improvement initiatives were in play at the same time (Sites 2, 3, 5 & 6). As one summarised about implant rationalisation:

> *…because I have been pushing hard based on the GIRFT report, but a lot of colleagues across the UK and certainly here haven't been very keen to adopt the changes. But then with the tariff reduction, we have had no choice, so the combination of the GIRFT report and the tariff reduction, we have now made those changes.* (Hybrid manager, Site 3)

On average, sites reported that there were 10 other improvement initiatives (range 8-17) taking place at the same time as GIRFT, all impacting orthopaedic care. All sites reported at least one local monitoring and improvement initiative (e.g. procedures rationalisation (site 2), infection minimisation (sites 1, 3-4), theatre utilisation (sites 1, 5-6), and procurement (sites 4, 6)); at least one relevant pathway or service redesign; and at least two national efficiency and improvement programmes (e.g. the Success Regime, Right Care, Cost Improvement Programme, and Vanguard status). These could, as in the example above, work together to provider leverage for improvement.

DISCUSSION

We have described the contextual factors that influenced adoption and implementation of GIRFT in orthopaedics and the ways in which these interacted to determine whether and how change took place. Contextual factors operated at three different levels, to varying degrees: the level of the orthopaedic team (e.g. how individuals interacted with the intervention); the wider Trust (e.g. competition for bed space and theatre time); and the health economy more broadly (e.g. requirements to form local networks). Although all sites had made improvements to care in recent years, very few were a direct consequence of their GIRFT visits. GIRFT did however contribute to some improvements, albeit indirectly. Some used GIRFT to provide evidence, which helped cement and formalise existing plans or processes; catalyse change; and leverage cross-professional support. Along with other concurrent initiatives, GIRFT also helped create an environment receptive to change. It was particularly impactful where its recommendations and pre-existing Trust priorities aligned. Unsurprisingly, where GIRFT measures were not a local priority because of more immediate demands – for example, financial and bed pressures – GIRFT was less likely to influence change.

This is part of the first independent evaluation of GIRFT.[9,16] Our mixed-method approach enabled us to provide a comprehensive and robust understanding of the programme and explore its impact from different perspectives. Previous similar evaluations tend to focus on quantitative analyses. We only focussed on GIRFT in orthopaedics but the findings about interactions between the intervention and multi-level contextual factors are transferable to other GIRFT clinical streams and other large-

For peer review only - http://bmjopen.bmj.com/site/about/guidelines.xhtml

scale improvement programmes. Although we have studied the process of implementation in a small number of sites (as is usual in this type of research), our case study approach led to a rich and detailed picture of changes as they occurred over time, in a variety of settings. At each site, we invited staff to interview who were present at the first GIRFT visit or otherwise knowledgeable about improvement work locally. We spoke to a range of professionals, including clinicians and managers, across all sites. However, it is possible that we have not captured all the changes that took place at Trusts, or all the factors that influenced implementation. It is also important to note that the recommendations to each Trust were bespoke, based on their performance data at the time, introducing a degree of heterogeneity into the content of the intervention. We collected empirical data between 2016 and 2019 while the programme was continuing to evolve, but we did not find any major changes during the evaluation period. Finally, the interviews took place several months after the first visits, creating a risk of recall bias.

Although there have been significant improvements in orthopaedic care nationally, many began prior to GIRFT. Changes observed over the past 10 years are likely attributable to both GIRFT and other concurrent initiatives, but it is not possible to determine their relative contributions. The additional impact of Trust visits was mixed.[9] The findings we describe here help explain why this is the case: where GIRFT aligned with pre-existing Trust priorities, staff were able to leverage the programme to support change. Where GIRFT measures were not a local priority, it had much less impact. In 2020, the GIRFT team published an internal evaluation[23] of the orthopaedic workstream, citing individual case studies that exemplified success. Our diverse, purposively selected range of sites facilitated cross-case comparison. This paper also adds to the wider literature about the role of context in healthcare improvement. To date, only a small number of studies have explicitly focused on interactions between contextual factors.[24] We have demonstrated that dynamic relationships within and between levels, rather than any one factor individually, were key in shaping the impact of a major national improvement programme.

Interviewees from all our sites argued that their deep-dive visit had not been a two-way dialogue, with limited opportunities for attendees to question and challenge. Many also reported that GIRFT did not acknowledge the importance of local organisational context. Both things negatively influenced the impact of GIRFT. Those designing and delivering future improvement programmes should consider how all three levels of context (macro, meso and micro) and the interactions that may occur between them could affect their intervention, rather than focusing on individual factors or levels in isolation. This is especially important for large scale (e.g. national) programmes where there is likely to be significant variation in local context. Case study research, with its small sample sizes, does not set out to create explanatory models for implementation. However, we highlight a number of contextual influences that are likely to be important, such as the nature and configuration of participating Trusts, and the presence or absence of external pressures, for example financial or regulatory challenges. Although some contextual factors can be modified by Trusts (for example, organisational cultures and leadership) many can not (for example, government policy) and, therefore, need to be taken into account by those implementing improvement programmes[24]

GIRFT is one of the largest improvement initiatives in the NHS. Following its introduction in orthopaedics, it was rolled out at substantial cost in over 40 other clinical areas, without independent evaluation. We have previously noted that changes to orthopaedic practice over the

For peer review only - http://bmjopen.bmj.com/site/about/guidelines.xhtml

past 10 years are likely attributable to both GIRFT and other concurrent initiatives.[9] Our findings here help explain why the additional impact of GIRFT visits to individual Trusts was mixed: although all case sites had made improvements to care, very few were directly attributed to GIRFT. A range of factors, operating at different levels, also influenced sites' ability to implement GIRFT recommendations. As well as early engagement with rigorous evaluation design, those delivering future improvement programmes should consider how their intervention sits in relation to the three levels of context (macro, meso and micro) and the dynamic interactions that may occur between them.

For peer review only

For peer review only - http://bmjopen.bmj.com/site/about/guidelines.xhtml

DECLARATIONS

Acknowledgements: We thank Patricia Hallam for administrative support for the study. We thank all the other members of the research team who contributed to other parts of this mixed methods evaluation. We thank the public members of the NIHR ARC North Thames Research Advisory Panel who commented on all aspects of the study and the professional stakeholders who gave their time to take part in interviews and provide relevant documentation. We thank the GIRFT programme team for providing lists of sites from which to recruit case sites anonymously.

Contributors: HB was principal investigator. HB and RR initiated the research. HB, NJF and RR designed the evaluation. SJ and JL were responsible for qualitative data collection; FA, JL, SJ, NJF and HB analysed the qualitative data. FA, JL, SJ, RR, NJF and HB drafted the manuscript and all authors contributed to reviewing and substantive revision. All authors approved the final version. All authors had full access to all the data in the study and accept responsibility to submit for publication. HB will act as guarantor.

Funding: This reports independent research funded by the National Institute for Health Research (NIHR) Applied Research Collaboration (ARC) North Thames (award/grant number is not applicable).

Disclaimer: This report is independent research funded by the National Institute for Health Research ARC North Thames. The views expressed in this publication are those of the authors and not necessarily those of the National Institute for Health Research or the Department of Health and Social Care.

Competing interests: All authors have completed the ICMJE uniform disclosure form at www.icmje.org/coi disclosure.pdf, and declare: FA, JL, SJ, HB, RM, RR, HB are funded in full or in part by the National Institute for Health Research ARC North Thames. RR and NJF are NIHR Senior Investigators; no financial relationships with any organisations that might have an interest in the submitted work in the previous 3 years; no other relationships or activities that could appear to have influenced the submitted work.

Ethics approval and consent to participate: This study involved human participants and was reviewed and approved by NHS North West - Liverpool East Research Ethics Committee (16/NW/0654). Before taking part, all participants gave informed consent to participate in the study and for be analysed and reported anonymously.

Provenance and peer review: Not commissioned; externally peer reviewed.

Data availability statement: Due to the professional sensitivity of these data, qualitative data are not publicly available. Informed consent did not extend to use of these data outside the study.

Consent for publication – Not applicable

Availability of data and materials – Consent was not given by participants to share their anonymised transcripts.

For peer review only

For peer review only - http://bmjopen.bmj.com/site/about/guidelines.xhtml

ORCID iDs:

Fiona Aspinal http://orcid.org/0000-0003-3170-7570

Jean Ledger http://orcid.org/0000-0003-2523-7971

Sarah Jasim http://orcid.org/0000-0003-3940-6350

Raj Mehta http://orcid.org/0000-0002-4003-530X

Rosalind Raine http://orcid.org/0000-0003-0904-749X

Naomi J Fulop http://orcid.org/0000-0001-5306-6140

Helen Barratt http://orcid.org/0000-0002-1387-137X

For peer review only

REFERENCE LIST

1. Culliford D, Maskell J, Judge A, et al. Future projections of total hip and knee arthroplasty in the UK: results from the UK Clinical Practice Research Datalink. *Osteoarthritis and Cartilage* 2015;23(4):594-600. doi: 10.1016/j.joca.2014.12.022
2. OECD. Hip and knee replacement: Health at a Glance 2019. OECD Indicators. Paris: OECD Publishing 2019.
3. Gunsche JL, Pilz V, Hanstein T, et al. The variation of arthroplasty procedures in the OECD Countries: analysis of possible influencing factors by linear regression. *Orthopedic Reviews* 2020;12(3):118-23. doi: 10.4081/or.2020.8526
4. Abdelaal MS, Restrepo C, Sharkey PF. Global Perspectives on Arthroplasty of Hip and Knee Joints. *Orthopedic Clinics of North America* 2020;51(2):169-+. doi: 10.1016/j.ocl.2019.11.003
5. Carr A, Smith JA, Camaradou J, et al. Growing backlog of planned surgery due to covid-19. *BMJ* 2021;372:n339.
6. Briggs T. Getting it right first time. *Improving the quality of orthopaedic care within the National Health Service in England* 2012
7. Briggs T. A national review of adult elective orthopaedic services in England. *Getting it right first time* 2015;7
8. GIRFT. Getting it Right first Time 2022 [Available from: https://www.gettingitrightfirsttime.co.uk/girft-methodology/. accessed 16/04/2022 2022.
9. Barratt H, Hutchings A, Pizzo E, et al. Mixed methods evaluation of the Getting it Right First Time programme in elective orthopaedic surgery in England: an analysis from the National Joint Registry and Hospital Episode Statistics. *BMJ open* 2022;12(6):e058316.
10. Robert G, Fulop N. The role of context in successful improvement. *Perspectives on context A selection of essays considering the role of context in successful quality improvement London: Health Foundation* 2014;31
11. Horton T, Illingworth J, Warburton W. The spread challenge. *UK: Health Foundation* 2018
12. Dixon-Woods M, McNicol S, Martin G. Ten challenges in improving quality in healthcare: lessons from the Health Foundation's programme evaluations and relevant literature. *Bmj Quality & Safety* 2012;21(10):876-84. doi: 10.1136/bmjqs-2011-000760
13. Currie G, Spyridonidis D. Sharing leadership for diffusion of innovation in professionalized settings. *Human Relations* 2019;72(7):1209-33. doi: 10.1177/0018726718796175
14. Fulop NJ, Ramsay AIG. How organisations contribute to improving the quality of healthcare (vol 365, l1773, 2019). *Bmj-British Medical Journal* 2019;366 doi: 10.1136/bmj.l4496
15. Burgoyne J, Williams S, Walmsley J. Evaluation of the Shared Leadership for Change Award Scheme. *London: The Health Foundation* 2009
16. Barratt H, Turner S, Hutchings A, et al. Mixed methods evaluation of the Getting it Right First Time programme - improvements to NHS orthopaedic care in England: study protocol. *BMC Health Services Research* 2017;17(1):71.
17. Yin RK. Case study research: Design and methods. Thousand Oaks. *Sage Young, LC and Wilkinson, IR (1989) The role of trust and co-operation in marketing channels: a preliminary study European Journal of Marketing* 2003;23(2):109-22.
18. Yin RK. Validity and generalization in future case study evaluations. *Evaluation* 2013;19(3):321-32.
19. Bowling A. Research Methods in Health. Open University Press. *Maidenhead, England* 2002
20. Braun V, Clarke V. Contextualism. thematic analysis a practical guide: London: Sage Publications, 2022.
21. Lingard L. Beyond the default colon: Effective use of quotes in qualitative research. *Perspectives on Medical Education* 2019;8(6):360-64. doi: 10.1007/s40037-019-00550-7
22. British Orthopaedic Association. BOA Professional Guidance to Implement Getting it Right First Time in England. London: BOA, 2016.

For peer review only - http://bmjopen.bmj.com/site/about/guidelines.xhtml

23. GIRFT. Reflecting on success and reinforcing improvement: a follow-up on the GIRFT national specialty report on orthopaedics. London: GIRFT, 2020.
24. Fulop N, Robert G. Context for successful quality improvement: Health Foundation 2015.

For peer review only

For peer review only - http://bmjopen.bmj.com/site/about/guidelines.xhtml

Standards for Reporting Qualitative Research (SRQR)*

http://www.equator-network.org/reporting-guidelines/srqr/

Title and abstract

	Page/line no(s).
**Title** - Concise description of the nature and topic of the study Identifying the study as qualitative or indicating the approach (e.g., ethnography, grounded theory) or data collection methods (e.g., interview, focus group) is recommended	1
**Abstract** - Summary of key elements of the study using the abstract format of the intended publication; typically includes background, purpose, methods, results, and conclusions	2

Introduction

**Problem formulation** - Description and significance of the problem/phenomenon studied; review of relevant theory and empirical work; problem statement	4
**Purpose or research question** - Purpose of the study and specific objectives or questions	5

Methods

**Qualitative approach and research paradigm** - Qualitative approach (e.g., ethnography, grounded theory, case study, phenomenology, narrative research) and guiding theory if appropriate; identifying the research paradigm (e.g., postpositivist, constructivist/ interpretivist) is also recommended; rationale**	5
**Researcher characteristics and reflexivity** - Researchers' characteristics that may influence the research, including personal attributes, qualifications/experience, relationship with participants, assumptions, and/or presuppositions; potential or actual interaction between researchers' characteristics and the research questions, approach, methods, results, and/or transferability	
**Context** - Setting/site and salient contextual factors; rationale**	5
**Sampling strategy** - How and why research participants, documents, or events were selected; criteria for deciding when no further sampling was necessary (e.g., sampling saturation); rationale**	5 & 6
**Ethical issues pertaining to human subjects** - Documentation of approval by an appropriate ethics review board and participant consent, or explanation for lack thereof; other confidentiality and data security issues	19
**Data collection methods** - Types of data collected; details of data collection procedures including (as appropriate) start and stop dates of data collection and analysis, iterative process, triangulation of sources/methods, and modification of procedures in response to evolving study findings; rationale**	6

For peer review only

**Data collection instruments and technologies** - Description of instruments (e.g., interview guides, questionnaires) and devices (e.g., audio recorders) used for data collection; if/how the instrument(s) changed over the course of the study	6
**Units of study** - Number and relevant characteristics of participants, documents, or events included in the study; level of participation (could be reported in results)	7 & 8
**Data processing** - Methods for processing data prior to and during analysis, including transcription, data entry, data management and security, verification of data integrity, data coding, and anonymization/de-identification of excerpts	6
**Data analysis** - Process by which inferences, themes, etc., were identified and developed, including the researchers involved in data analysis; usually references a specific paradigm or approach; rationale**	6 & 7
**Techniques to enhance trustworthiness** - Techniques to enhance trustworthiness and credibility of data analysis (e.g., member checking, audit trail, triangulation); rationale**	5, 6 & 19

Results/findings

**Synthesis and interpretation** - Main findings (e.g., interpretations, inferences, and themes); might include development of a theory or model, or integration with prior research or theory	8-14
**Links to empirical data** - Evidence (e.g., quotes, field notes, text excerpts, photographs) to substantiate analytic findings	8-14

Discussion

**Integration with prior work, implications, transferability, and contribution(s) to the field -** Short summary of main findings; explanation of how findings and conclusions connect to, support, elaborate on, or challenge conclusions of earlier scholarship; discussion of scope of application/generalizability; identification of unique contribution(s) to scholarship in a discipline or field	14-15
**Limitations** - Trustworthiness and limitations of findings	3 & 15

Other

**Conflicts of interest** - Potential sources of influence or perceived influence on study conduct and conclusions; how these were managed	19
**Funding** - Sources of funding and other support; role of funders in data collection, interpretation, and reporting	19

*The authors created the SRQR by searching the literature to identify guidelines, reporting standards, and critical appraisal criteria for qualitative research; reviewing the reference lists of retrieved sources; and contacting experts to gain feedback. The SRQR aims to improve the transparency of all aspects of qualitative research by providing clear standards for reporting qualitative research.

For peer review only - http://bmjopen.bmj.com/site/about/guidelines.xhtml

**The rationale should briefly discuss the justification for choosing that theory, approach, method, or technique rather than other options available, the assumptions and limitations implicit in those choices, and how those choices influence study conclusions and transferability. As appropriate, the rationale for several items might be discussed together.

Reference:

O'Brien BC, Harris IB, Beckman TJ, Reed DA, Cook DA. **Standards for reporting qualitative research: a synthesis of recommendations.** *Academic Medicine*, Vol. 89, No. 9 / Sept 2014 DOI: 10.1097/ACM.0000000000000388

For peer review only

For peer review only - http://bmjopen.bmj.com/site/about/guidelines.xhtml

Implementation of the national Getting it Right First Time orthopaedic programme in England: a qualitative case study analysis.

Journal:	*BMJ Open*
Manuscript ID	bmjopen-2022-066303.R1
Article Type:	Original research
Date Submitted by the Author:	02-Feb-2023
Complete List of Authors:	Aspinal, Fiona; University College London, Department of Applied Health Research<br>Ledger, Jean; University College London, Department of Applied Health Research<br>Jasim, Sarah; The London School of Economics and Political Science, Care Policy and Evaluation Centre<br>Mehta, Raj; University College London, NIHR ARC North Thames Research Advisory Panel, Department of Applied Health Research<br>Raine, Rosalind; University College London, Department of Applied Health Research<br>Fulop, Naomi; University College London, Department of Applied Health Research<br>Barratt, Helen; University College London, Department of Applied Health Research
**Primary Subject Heading**:	Qualitative research
Secondary Subject Heading:	Health services research
Keywords:	Quality in health care < HEALTH SERVICES ADMINISTRATION & MANAGEMENT, Adult orthopaedics < ORTHOPAEDIC & TRAUMA SURGERY, QUALITATIVE RESEARCH, Orthopaedic & trauma surgery < SURGERY

SCHOLARONE™
Manuscripts

I, the Submitting Author has the right to grant and does grant on behalf of all authors of the Work (as defined in the below author licence), an exclusive licence and/or a non-exclusive licence for contributions from authors who are: i) UK Crown employees; ii) where BMJ has agreed a CC-BY licence shall apply, and/or iii) in accordance with the terms applicable for US Federal Government officers or employees acting as part of their official duties; on a worldwide, perpetual, irrevocable, royalty-free basis to BMJ Publishing Group Ltd ("BMJ") its licensees and where the relevant Journal is co-owned by BMJ to the co-owners of the Journal, to publish the Work in this journal and any other BMJ products and to exploit all rights, as set out in our licence.

The Submitting Author accepts and understands that any supply made under these terms is made by BMJ to the Submitting Author unless you are acting as an employee on behalf of your employer or a postgraduate student of an affiliated institution which is paying any applicable article publishing charge ("APC") for Open Access articles. Where the Submitting Author wishes to make the Work available on an Open Access basis (and intends to pay the relevant APC), the terms of reuse of such Open Access shall be governed by a Creative Commons licence – details of these licences and which Creative Commons licence will apply to this Work are set out in our licence referred to above.

Other than as permitted in any relevant BMJ Author's Self Archiving Policies, I confirm this Work has not been accepted for publication elsewhere, is not being considered for publication elsewhere and does not duplicate material already published. I confirm all authors consent to publication of this Work and authorise the granting of this licence.

For peer review only

Implementation of the national Getting it Right First Time orthopaedic programme in England: a qualitative case study analysis.

*Fiona Aspinal, Department of Applied Health Research, University College London, 1-19 Torrington Place, London, WC1E 7HB, UK f.aspinal@ucl.ac.uk

Jean Ledger, Department of Applied Health Research, University College London, UK

Sarah Jasim, Care Policy and Evaluation Centre (CPEC), London School of Economics and Political Science, London, UK

Raj Mehta, Patient Advisor, NIHR ARC North Thames Research Advisory Panel, Department of Applied Health Research, University College London, UK

Rosalind Raine, Department of Applied Health Research, University College London, London, UK

Naomi Fulop, Department of Applied Health Research, University College London, London, UK

Helen Barratt, Department of Applied Health Research, University College London, London, UK

*corresponding author

Word count: 4554 (406 are quotes)

For peer review only - http://bmjopen.bmj.com/site/about/guidelines.xhtml

ABSTRACT

Objective: To describe the implementation and impact of the Getting it Right First Time (GIRFT) national orthopaedic improvement programme at the level of individual National Health Service (NHS) Trusts.

Design: Qualitative case studies conducted at six NHS Trusts, as part of a mixed methods evaluation of GIRFT.

Setting: NHS elective orthopaedic surgery in England.

Participants: 59 NHS staff

Intervention: Improvement bundle, including bespoke routine performance data and improvement recommendations for each organisation, delivered via 'deep-dive' visits to NHS Trusts by a senior orthopaedic clinician.

Results: Although all case study sites had made improvements to care, very few of these were reportedly a direct consequence of GIRFT. A range of factors, operating at three different levels, influenced their ability to implement GIRFT recommendations: at the level of the orthopaedic team (micro - e.g. how individuals perceived the intervention); the wider Trust (meso - e.g. competition for theatre/bed space); and the health economy more broadly (macro - e.g. requirements to form local networks). Some sites used GIRFT evidence to support arguments for change which helped cement and formalise existing plans. However, where GIRFT measures were not a Trust priority because of more immediate demands– for example, financial and bed pressures – it was less likely to influence change.

Conclusion: Dynamic relationships between the different contextual factors, within and between the three levels, can impact the effectiveness of a large-scale improvement intervention and may account for variations in implementation outcomes in different settings. When designing an intervention, those leading future improvement programmes should consider how it sits in relation to these three contextual levels and the interactions that may occur between them.

For peer review only - http://bmjopen.bmj.com/site/about/guidelines.xhtml

Strengths and limitations of this study

- This work is part of the first independent evaluation of the high profile, national improvement programme, Getting it Right First Time (GIRFT).
- Observing 'deep-dive' visits to Trusts by the GIRFT team, reviewing local documentation, and interviewing clinicians and managers, enabled us to explore in-depth the factors that influenced the implementation and impact of GIRFT at a local level.
- Although we only studied the process of implementation at six NHS provider Trusts, our case study approach created a rich and detailed picture of changes over time, in a variety of settings.
- We spoke to a range of clinicians and managers at each site, but it is possible that we may not have captured all the improvements at each site, not least because the interviews took place several months after the first GIRFT visits creating a risk of recall bias.

For peer review only

For peer review only - http://bmjopen.bmj.com/site/about/guidelines.xhtml

BACKGROUND

Orthopaedic procedures such as total hip and total knee replacement are among the commonest surgical procedures in the UK[1] and worldwide.[2,3] Demand is likely to increase given ageing populations and the associated increased need for arthroplasty surgery. This is likely to have a significant impact on health systems globally[4] including the National Health Service (NHS) in England, which already faces a backlog of planned surgery due to the COVID-19 pandemic.[5]

'Getting it Right First Time' (GIRFT) is one of the largest improvement programmes in the NHS. It began in orthopaedics in 2012.[6] Following government investment of over £62m, it now operates in 44 different specialties or clinical workstreams across England.[7] GIRFT aims to draw clinicians and managers together to reduce unwarranted variation, improve outcomes and maximise efficiency.[7] In orthopaedics, this includes changing practice (e.g. prosthesis choice); organisational processes (e.g. procurement, length of stay); and service delivery (e.g. ring-fenced beds).[8]Clinical leadership is fundamental: GIRFT was established by a senior surgeon, who leads the orthopaedic workstream and chairs the wider programme. Each workstream is chaired by a clinical lead from the relevant specialty. The programme includes components operating locally (i.e. at NHS provider Trusts) and nationally (i.e. across England). Local components include 'deep dive' visits to Trusts, while national components include reports describing how variations can be addressed. Before each visit, Trusts are sent a 'datapack,' collated by GIRFT, describing their performance on over 100 variables, drawn from sources including Hospital Episode Statistics and the National Joint Registry. Datapacks cover use of evidence-based procedures and costs, and facilitate comparison to national and peer group averages. Discussion at the visits is driven by the datapack and tailored to the Trust. Attendees, comprising clinicians, managers and other relevant professionals, identify where improvements could be made. Although nationally there have been improvements in orthopaedic care over the last decade, these began before GIRFT. Other concurrent initiatives also targeted similar outcomes (e.g. Lord Carter's review of NHS efficiency and NHS Right Care), so it is not possible to quantify the impact of GIRFT in isolation from other national programmes. Estimations of the additional impact of GIRFT deep dive Trusts visits, found a mix of positive and negative effects that were generally small compared with overall improvement trends.[9]

It is increasingly recognised that, at an organisational level (e.g. within individual NHS Trusts), the impact of an intervention such as GIRFT, is shaped by multiple, contextual factors.[10] Context is defined as the underlying systems, culture and circumstances of the environment in which an intervention is implemented.[11] Contextual factors operate at three levels: micro (individual); meso (organisational); and macro-level (external/policy). At the micro-level of individuals in healthcare settings trying to implement change, collaboration must be fostered to facilitate change[12] and clinical leaders are often able to exert particular influence because of their professional credibility.[13] At the meso-level, health care organisations themselves play a central role in improvement.[14] Relevant factors may include processes (e.g. how strategy is developed); leadership (e.g. how leaders engage with their external context), and underlying features (e.g. leadership stability), all of which vary between different settings.[10] Outer 'macro-level' context refers to influences beyond the organisation, such as government policy and regulation, which can have a strong influence on how organisations approach improvement.[15] Whilst the intervention and local context interact to determine whether and how improvement occurs, it is increasingly

For peer review only - http://bmjopen.bmj.com/site/about/guidelines.xhtml

recognised that interactions between the three levels of context can also serve to effect improvement or confound it.[10]

Although the influence of context on improvement is widely acknowledged, understanding of its role is still limited.[14] In this paper, we describe the implementation and impact of GIRFT at the level of individual Trusts. We explore both the contextual factors that influenced implementation and the ways in which the interaction of factors operating at different levels determined whether and how change took place. In doing so, we contribute to knowledge about how context influences how national improvement programmes effect change, and to inform the design and development of context-sensitive interventions in the future. This research was part of a wider mixed methods evaluation of the GIRFT orthopaedic programme.[9,16]

DESIGN

We used a case study approach to examine the different ways Trusts implemented GIRFT and how this influenced the impact of the programme locally. Case studies allow complex phenomena such as improvement to be studied in-depth where there are multiple variables and dynamics intersecting to influence change. The unit of the case study (here, organisations providing orthopaedic services) can be considered with respect to their internal context (such as professional leadership and organisational processes), and interactions between the case study and other external factors (e.g. national trends or policy). . Undertaking multiple case studies facilitates comparison and can increase the transferability of data to other situations and contexts.[17,18] Rather than data saturation, we sought breadth of perspectives. Decisions about the number of sites/interviews involved ongoing discussion within the research teams and a trade-off between research resources available and maximising the range of sites and the availability of interviewees (many of whom were front-line clinicians).

Case selection

We identified six case study sites, all NHS Trusts participating in the GIRFT orthopaedic programme. We selected sites purposively to represent a spread of hospital types (district general and teaching hospitals); different levels of performance on GIRFT metrics and engagement with the GIRFT programme at the time of recruitment; and settings (region of England; rural versus urban).

This was an independent evaluation of the GIRFT programme. However, the GIRFT programme team facilitated our understanding of the programme by providing updates about programme changes, dates of deep-dive visits across Trusts in England, and access to key programme documentation. In July 2016, the GIRFT programme team provided a list of Trusts grouped according to their initial qualitative assessment of a) performance on GIRFT metrics at that time and b) extent to which they perceived managers and clinicians were committed to implementing change together. From this, we identified an initial list of six potential sites to meet the wider criteria and emailed the clinical lead for trauma and orthopaedics at each. Four responded positively, but we were unable to secure engagement at the other two – one high engagement and one low engagement site. We returned to the list and identified then recruited, two similar Trusts to replace to those in our initial shortlist.

with the information provided by the GIRFT team to identify Trusts' level of engagement with the GIRFT programme at the early stage of implementation enabled us to draw on their knowledge of

For peer review only - http://bmjopen.bmj.com/site/about/guidelines.xhtml

orthopaedic performance and key individuals at Trusts. However, the GIRFT team had no input into the final selection of sites (nor any other research decisions). The independence of this research from the GIRFT programme team was made clear to participating sites. Data collection was only carried out by members of the research team. The GIRFT programme team were not informed which sites or individuals took part, and all were anonymised throughout the study.

METHODS

Case study research draws on a variety of data sources to construct a detailed picture of phenomena (in this case, implementation and impact of the GIRFT programme) in their 'natural setting'.[19] In each of the six sites, data were collected via non-participant observation of GIRFT visits, semi-structured telephone and in-person interviews, and collection and analysis of local literature/documents. Two researchers (JL & SJ) took the lead at different case sites (JL was the lead in four sites and SJ in two). Data were collected between October 2016 and May 2019. This long data collection period facilitated us conducting two rounds of interviews per case site and enabled the second of these, as far as possible, to coincide with Trusts' follow-up GIRFT deep-dive visits.

Interviews

We (JL & SJ) conducted a round of semi-structured interviews with orthopaedic surgeons, managers and other key staff who were present at the first Trust visit and/or knowledgeable about improvement work locally. The interviews covered: (i) organisational engagement with the GIRFT programme and the reasons for this; (ii) practice and service delivery changes, particularly those concerning key GIRFT metrics such as ring-fenced beds, low volume surgeons, fixation methods and procurement; and (iii) perceptions of the impact of GIRFT on any changes. The topic guide (see supplementary file) was piloted prior to the interviews and refined iteratively as the study progressed to take account of emerging findings. A second round of interviews was conducted around the time of the Trusts' follow-up deep-dive visit. Similar topics (see supplementary file) were covered, with a focus on change over time including implementation of GIRFT recommendations, and continued engagement with the programme. Some people were interviewed at both time points and some at a single time point. All interviewees gave informed consent. Interviews lasted between 20 minutes and 1 hour and were digitally recorded for professional transcription in full.

Observation of deep-dive visits

Researchers (JL & SJ) attended and observed all GIRFT orthopaedic visits that took place at case study sites during the data collection period. This provided an understanding of the data being presented to the sites by the GIRFT team; the local context; and local receptiveness/engagement with the GIRFT programme. Researchers kept field journals for recording notes about attendee behaviours and interactions during meeting observations. They also attended deep-dive visits at non-case study Trusts and GIRFT events to better understand the wider programme and its delivery. This helped obscure the identities of participating sites from the GIRFT team. Consent to attend deep-dive visits was granted by the clinician leading the GIRFT programme in the case site, who asked attendees to indicate if they did not consent to researchers attending and taking notes. Consent was given at all case deep-dive visits. Researchers did not take fieldnotes when attendees indicated that a discussion point was confidential.

For peer review only - http://bmjopen.bmj.com/site/about/guidelines.xhtml

Local documents

We collated documents from each site, including GIRFT data-packs and presentations, Trust annual reports, and local documents about, for example, other improvement programmes or plans for local musculo-skeletal or orthopaedic care pathways. These provided information about local approaches to improvement; competing/complementary priorities; the local population; Trust services and performance; and wider strategic decisions.

Patient and public involvement

Patient and public advice on the design and conduct of this research was provided via the National Institute for Health and Care research (NIHR) Applied Research Collaboration (ARC) North Thames' Research Advisory Panel. A patient representative (RM) was a core member of the research advisory group throughout the project.

DATA ANALYSIS

Interviews were subjected to thematic analysis.[20] Data from documents and observations were reviewed to help interpret and contextualise interview data. Observation notes were included in thematic analysis where appropriate.

JL and SJ produced detailed, narrative case portraits for each case site, drawing on case transcripts, observation fieldnotes and documents. FA & HB read all data across all sites and, together with JL & SJ, held regular cross-case comparison analysis meetings to identify key themes across the data. Because each researcher has different case site and disciplinary expertise, we were able to identify and balance each individual's assumptions about the data. Together, we iteratively identified and determined the parameters of themes. FA then undertook line-by-line coding, applying these themes to data across all the sites. Findings were reviewed and confirmed by SJ, JL and HB.

The initial coding frame combined inductive and deductive approaches. It focussed on whether and how sites engaged with GIRFT; reported 'improvements' in care; and the factors affecting this. Cross-case comparison drew on the interaction between different levels of context (micro, meso and macro) to help explain how Trusts responded to a large-scale quality improvement programme.

Quotations used in the findings are illustrative of the points being made and representative of the wider patterns in the data.[21]

FINDINGS

Information about participating sites, interviewees and data sources are presented before the findings from the case study analysis.

The six case sites represent a spread of Trust types, rurality, level of engagement with the programme and geographical region. To protect the anonymity of sites, we have summarised their characteristics data in Table 1.

For peer review only - http://bmjopen.bmj.com/site/about/guidelines.xhtml

For peer review only

Table 1: Participating case sites

Criteria	Category	Counts
**Type of Trust**	District General	3
	Teaching	3
**Setting[1]**	Rural	2
	Urban	3
	Mixed	1
**Level of engagement with the GIRFT programme at time of recruitment**	High	2
	Medium	2
	Low	2
**Region of England**	East of England	1
	London	1
	North East & Yorkshire	1
	South East	2
	South West	1

[1]defined by interviewees and/or local public health annual reports.

We interviewed 59 key stakeholders across the six Trusts, including orthopaedic surgeons (some of whom were also managers), other clinical staff, and hospital managers (some of whom retained a clinical role), including at least one representatives of Trusts' executive leadership team in each site. Those interviewees with a joint clinical and managerial role, are referred to here as a 'hybrid manager'. Forty-six people were interviewed at Time 1 and 13 at time two. Some people preferred to be interviewed together with one or more colleagues: in Site 1, one interview was conducted with two people and another with three, and in Site 2 one of the interviews included two people. Table 2 shows the interviewees at each time point.

For peer review only

Table 2: Interviewees per case site

	Time	Surgeon	Nurse	Hybrid*	Manager	Commissioner	Total per time point	Total per site
**Site1**	T1	1		5	2	3	11	**14**
	T2	1		1		1	3	
**Site2**	T1	2		1	5		8	**11**
	T2	1			2		3	
**Site3**	T1	3	1	1			5	**7**
	T2			1	1		2	
**Site4**	T1	2		3	2		7	**9**
	T2	2					2	
**Site5**	T1	6			1		7	**8**
	T2				1		1	
**Site6**	T1	2		2	4		8	**10**
	T2				2		2	
**Total**		**20**	**1**	**14**	**20**	**4**	**T1 = 46**<br>**T2 = 13**	**59**

*Hybrid includes clinician/managers

Case study analysis

Before each 'deep dive' visit, Trusts were sent a 'datapack' from the national GIRFT team describing their performance on over 100 variables. Each visit was led by the GIRFT orthopaedic clinical lead. Discussion was driven by the datapack, so recommendations were specific to each Trust. Here, we explore first the factors that affected the adoption and implementation of GIRFT, including micro, meso and macro-level contextual influences. We then describe changes made to orthopaedic care at case study sites and the extent to which these were attributable to GIRFT.

Micro-level contextual factors

At the individual level, managers and clinicians who were interviewed demonstrated good understanding of GIRFT and its aims. They described a variety of routes through which they first heard about the programme, including via the British Orthopaedic Association (BOA); colleagues; the initial GIRFT report;[6] and attending deep-dive visits at their Trust. However, many claimed that neither the underlying concepts nor delivery approach were novel:

> *Well, I suppose I became aware of the concepts that underpin GIRFT before GIRFT existed.* (Hybrid professional, Site 6)

Overall, interviewees across all sites supported what the programme sought to do – reducing unwarranted variation, maximising quality and promoting efficiency – and this made them more open to adopting the programme. As one participant said:

> *Personally, I thought "At last!" It is fantastic.* (Hybrid professional, Site 3)

Two major factors influenced how individuals perceived the intervention itself. The first was how they viewed the programme as a whole. At three sites, interviewees argued that their deep-dive

For peer review only - http://bmjopen.bmj.com/site/about/guidelines.xhtml

For peer review only

visits focussed on costs rather than quality. In one, for example, a senior manager argued that while the Trust always emphasised quality over cost, their deep-dive visit focussed on:

> *…money and efficiency and how many patients you can get through and how cheap you can buy prostheses for that work…* (Hybrid manager, Site 4).

In the sites where GIRFT was perceived to focus more on costs, there was low engagement, especially from surgeons, although in one site (Site 6) this perceived cost focus triggered high engagement from managers.

The second factor was the approach taken by the GIRFT clinical lead. While almost all interviewees acknowledged his expertise, some described how his particular approach in deep-dive visits affected their opinion of, and willingness to engage with, the programme. Some admired his style for '*not tak[ing] any of [the surgeons'] rubbish*' (Manager, Site 2), others found him '*supportive'* (Surgeon, Site 5) and '*very compelling*' (Hybrid manager, Site 6). However, others considered him '*adversarial'* (Manager, Site 6) and '*patronising'* (Manager, Site 4).

Those interviewees aligned with the latter views expressed more reluctance to actively engage with GIRFT. Where these individuals had power to facilitate or resist engagement with the programme, these views negatively impacted adoption and implementation locally.

Equally, interviewees from all six sites argued that their visits had not taken the form of a two-way dialogue – a finding supported by the observations of the deep-dive visits (although changes to the deep-dive visit format in two sites later in the data collection period were observed to provide an opportunity for two-way information exchange) - limiting opportunities for attendees to question and challenge data, approaches and metrics. Consequently, at least some disengaged with the process, risking delivery of the programme. As one manager noted:

> *I'm sure [the surgeons] would all value [the GIRFT] process and the information, but it has to be delivered in a way that's open to discussion and negotiation and mutual understanding.* (Manager, Site 4).

GIRFT is now being rolled out to over 40 other specialities, each of which has separate clinical lead. Interviewees who had attended visits in other specialities described different delivery approaches, which enabled two-way dialogue and a more flexible approach. This, they suggested, helped maintain their engagement.

The need for flexibility in implementing GIRFT recommendations was also highlighted. For example, interviewees felt that there was an expectation that Trusts would change practice simply to comply with GIRFT priorities, even where their outcomes were already above average and there was no cost benefit to derive from the change. For example, at Sites 1, 2, 3 and 5, at least some surgeons were reluctant to switch to cemented fixation for all hip arthroplasty patients over 65 because it did not allow for surgeon responsiveness (site 5) or consider the financial or wider organisational consequences of the additional training that would be required for staff (site 1, 2, 3, 5).

For peer review only - http://bmjopen.bmj.com/site/about/guidelines.xhtml

The BOA's guidance[22] about implementing GIRFT recognised the need for some flexibility, and was welcomed by interviewees:

> *Everybody in orthopaedics thinks that GIRFT is a good idea, but not everybody agrees with everything [the programme lead] says, so the [BOA] implementation document was a way of making [it] palatable to the wider profession*. (Hybrid manager, Site 3).

Meso-level contextual factors

At an organisational level, four of the sites had a long-embedded culture of quality improvement, including engaging with external quality initiatives in orthopaedics and across the Trust more widely. These Trusts already had internal processes for analysing performance data to inform service delivery and practice:

> *…the staff are very pro-department and the Trust and they want to make improvements and make the best journey and the best outcomes for our patients. So they do come up with ideas of how we can improve. The consultants are also very proactive with regards to what improvements can be made and how the team can be best set up to get the best outcomes for the patients.* (Lead nurse, Site 1)

In several of the case sites, Trust managers commented that engagement in the GIRFT programme was useful because it provided a '*free, independent quality audit*' (e.g. Manager, Site 4) and '*benchmarking*' (e.g. Surgeon, Site 4). More importantly GIRFT analysed more local data than some had capacity or resources to analyse internally. Already being open to instigating evidence-informed change did not always lead to GIRFT-instigated change, but it could catalyse change processes so that results were experienced more quickly. For example, in case site 3, GIRFT was used to expedite existing plans to streamline implant selection.

Nevertheless, across the sites, there was a feeling that GIRFT recommendations did not acknowledge the impact that Trust context could have on change. This included how the physical structure of a Trust could act as a barrier to improvement, even when individuals were convinced change was needed. For example, some interviewees argued that it was easier for multi-site orthopaedic services to separate trauma and elective care and, in turn, to ring-fence beds. In contrast, single-site Trusts often struggled to set aside beds for elective orthopaedics, especially during winter months:

> *So, like ring-fenced elective beds, that's an absolute critical requirement and recommendation of the GIRFT project. [Programme lead] talks about it a lot. And we all agree with that, but we still are unable to ring-fence elective orthopaedic beds because we are a massive hospital, an acute Trust, and lots of people are dying of flu and so on and so on. Those beds are needed to house the dying* [elderly] *in the winter.* (Surgeon, Site 5)

For peer review only - http://bmjopen.bmj.com/site/about/guidelines.xhtml

GIRFT recommendations linked to theatre capacity and bed usage, such as increasing the number of orthopaedic procedures per session or repatriating surgery from Any Qualified Providers (AQP), also encountered barriers. However, these were more about organisational complexity than organisational configuration. For example, interviewees talked about needing to *'vy[e] with urology, colorectal, vascular and all the other specialities'* (Hybrid manager, Site 6) to access theatre time, as well as negotiate bed availability across wards and arrange appropriate staffing.

Interviewees also noted that the complex planning needed to facilitate improvement in a busy acute Trust, means that change takes time. For example, interviewees in Site 6 described it taking eight months for ring-fenced beds to be implemented after Trust management had agreed because of other Trust priorities, space and bed demand-management, and associated cross-speciality negotiations (Surgeon, Site 6).

GIRFT's national peer benchmarking could, however, *'raise awareness'* (Surgeon, Site 2) and *'leverage support'* (Surgeon, Site 4) at a high level within Trusts that then catalyse improvement. As witnessed in Site 6, GIRFT benchmarking data prompted the site to institute ring-fenced beds, something that had been requested by clinicians, but had been on the backburner at the Trust for some years.

Macro-level contextual factors

Implementation of GIRFT recommendations by Trusts was also impacted by factors outside the organisation. For example, establishing networks to manage complex and low-volume procedures could be hampered by complex dynamics at a regional level. This was especially the case where Clinical Commissioning Group boundary and hospital catchment areas and/or the regional Sustainability and Transformation Partnership's (STP – now reorganised as Integrated Care Systems (ICS)) footprint were not aligned. In one site, GIRFT feedback was instrumental in a network being initiated, but this quickly broke down due to changing regional structures.

> [After the deep-dive visit] *we straight away looked at forming networks amongst the hospitals [to organise] the low volume procedures. Unfortunately, it's broken down… So, that's why, politically, it broke down, because we had to align with our own STPs. So, politically speaking, we had to withdraw from that and try and start up a new alliance with* [other Trusts] *in region*. (Surgeon, Site 2)

Impact

Interviewees were asked to describe any changes to orthopaedic care in recent years. Table 3 lists the changes that had been made in recent years and shows whether interviewees attributed this to GIRFT. Only two of the sites (Sites 1 & 2) confirmed that changes that had occurred were a direct result of GIRFT. Indeed, many indicated that their Trusts were working towards or had already addressed some of the GIRFT priorities before the programme started in 2012. This had occurred in response to organisational or regional performance management processes (Sites 1-6), organisational changes (e.g. leadership changes, Sustainability and Transformation Partnership (STP) requirements or CQC status) (Sites 1, 3 & 6) and/or preparing or delivering musculoskeletal pathway bids/contracts (Sites 1, 2, & 6). Thus, while many changes aligned with GIRFT, few were actually attributable to the programme directly (see Table 3).

For peer review only - http://bmjopen.bmj.com/site/about/guidelines.xhtml

Table 3: Changes reported by participants in sites and their attribution to GIRFT

	**Recent change attributed to GIRFT by study participants**			
**Sites**	**Fully**	**Partly**	**Possibly**	**Not**
**1**	Fixation method<br>Network development			
**2**	Fixation method<br>Network development			Procurement rationalisation<br>Arthroscopy reduction
**3**			Fixation method<br>Network development<br>Implant rationalisation	Length of Stay
**4**			Procurement rationalisation	Fixation method<br>Low-volume surgeons<br>Network development<br>Infection rates
**5**			Ring-fenced beds	Procurement rationalisation<br>Low-volume surgeons<br>Network development
**6**		Ring-fenced beds<br>Waiting list reduction		Low-volume surgeons<br>Network development

For peer review only

For peer review only - http://bmjopen.bmj.com/site/about/guidelines.xhtml

However, where change was already planned or in progress, some participants found the GIRFT programme and deep-dive visits useful for formalising plans (for example, formalising the existing regional MDT network in Site 5), catalysing action (network development in Site 1), and leveraging support for change (for example, surgeons leveraging management support for ring-fenced beds in Site 6).

In four of the sites, there was a question mark over the influence of GIRFT because interviewees were unsure of the relative contribution of the programme when other improvement initiatives were in play at the same time (Sites 2, 3, 5 & 6). As one summarised about implant rationalisation:

> *…because I have been pushing hard based on the GIRFT report, but a lot of colleagues across the UK and certainly here haven't been very keen to adopt the changes. But then with the tariff reduction, we have had no choice, so the combination of the GIRFT report and the tariff reduction, we have now made those changes.* (Hybrid manager, Site 3)

On average, sites reported that there were 10 other improvement initiatives (range 8-17) taking place at the same time as GIRFT, all impacting orthopaedic care. All sites reported at least one local monitoring and improvement initiative (e.g. procedures rationalisation (site 2), infection minimisation (sites 1, 3-4), theatre utilisation (sites 1, 5-6), and procurement (sites 4, 6)); at least one relevant pathway or service redesign; and at least two national efficiency and improvement programmes (e.g. the Success Regime, Right Care, Cost Improvement Programme, and Vanguard status). These could, as in the example above, work together to provider leverage for improvement.

DISCUSSION

We have described the contextual factors that influenced adoption and implementation of GIRFT in orthopaedics and the ways in which these interacted to determine whether and how change took place. Contextual factors operated at three different levels, to varying degrees: the level of the orthopaedic team (e.g. how individuals interacted with the intervention); the wider Trust (e.g. competition for bed space and theatre time); and the health economy more broadly (e.g. requirements to form local networks). Although all sites had made improvements to care in recent years, very few were a direct consequence of their GIRFT visits. GIRFT did however contribute to some improvements, albeit indirectly. Some used GIRFT to provide evidence, which helped to catalyse and formalise existing plans or processes, and to leverage cross-professional and organisational support. Along with other concurrent initiatives, GIRFT also helped create an environment receptive to change. It was particularly impactful where its recommendations and pre-existing Trust and professional priorities aligned. Unsurprisingly, where GIRFT measures were not a local priority because of more immediate demands – for example, financial and bed pressures – GIRFT was less likely to influence change. Programme leadership influenced acceptability and engagement with the programme both positively and negatively, and suggest that finding the right expertise and power balance between those leading and those implementing change is essential when promoting large-scale change.

For peer review only - http://bmjopen.bmj.com/site/about/guidelines.xhtml

This is part of the first independent evaluation of GIRFT.[9,16] Our mixed-method approach enabled us to provide a comprehensive and robust understanding of the programme and explore its impact from different perspectives. Previous similar evaluations tend to focus on quantitative analyses. We only focussed on GIRFT in orthopaedics but the findings about interactions between the intervention and multi-level contextual factors are transferable to other GIRFT clinical streams and other large-scale improvement programmes.

Although we have studied the process of implementation in a small number of sites (as is usual in this type of research), our case study approach led to a rich and detailed picture of changes as they occurred over time, in a variety of settings. The researchers collecting data (JL & SJ) were aware of the GIRFT programme team's initial engagement rating that we used to select Trusts. However, once case sites had been recruited, the focus of the evaluation was on participating Trusts'/individuals' perspectives on engagement with the GIRFT programme. As such, the initial assessment of engagement by the GIRFT team did not influence data collection or analysis. Although we identified common factors affecting engagement with GIRFT across the case sites, the small number of cases for each level of engagement means that we might not have identified the full range of factors that affect different, especially lower, levels of engagement with large-scale improvement programmes such as GIRFT. Two sites (one highly engaged and one with low engagement according to the GIRFT team's list) that were approached about taking part in the study did not respond to us and, consequently, we do not know their reasons for choosing not to participate in this evaluation. At each site, we invited staff to interview who were present at the first GIRFT visit or otherwise knowledgeable about improvement work locally. We spoke to a range of professionals, including clinicians and managers, across all sites. However, it is possible that we have not captured all the changes that took place at Trusts, or all the factors that influenced implementation. It is also important to note that the recommendations to each Trust were bespoke, based on their performance data at the time, introducing a degree of heterogeneity into the content of the intervention. We collected empirical data between 2016 and 2019 while the programme was continuing to evolve, but we did not find any major changes during the evaluation period. Finally, the interviews took place several months after the first visits, creating a risk of recall bias.

Although there have been significant improvements in orthopaedic care nationally, many began prior to GIRFT. Changes observed over the past 10 years are likely attributable to both GIRFT and other concurrent initiatives, but it is not possible to determine their relative contributions. The additional impact of Trust visits was mixed.[9] The findings we describe here help explain why this is the case: where GIRFT aligned with pre-existing Trust and professional priorities, staff were able to leverage the programme to support change. Where GIRFT measures were not a local priority, it had much less impact. In 2020, the GIRFT team published an internal evaluation[23] of the orthopaedic workstream, citing individual case studies that exemplified success. Our diverse, purposively selected range of sites facilitated cross-case comparison. This paper also adds to the wider literature about the role of context in healthcare improvement. To date, only a small number of studies have explicitly focused on interactions between contextual factors.[24] We have demonstrated that dynamic relationships within and between levels, rather than any one factor individually, were key in shaping the impact of a major national improvement programme.

For peer review only - http://bmjopen.bmj.com/site/about/guidelines.xhtml

Interviewees from all our sites argued that their deep-dive visit had not been a two-way dialogue, with limited opportunities for attendees to question and challenge. Many also reported that GIRFT did not acknowledge the importance of local organisational context. Both things negatively influenced the impact of GIRFT. Those designing and delivering future improvement programmes should consider how all three levels of context (macro, meso and micro) and the interactions that may occur between them could affect their intervention, rather than focusing on individual factors or levels in isolation. This is especially important for large scale (e.g. national) programmes where there is likely to be significant variation in local context. Case study research, with its small sample sizes, does not set out to create explanatory models for implementation. However, we highlight a number of contextual influences that are likely to be important, such as the nature and configuration of participating Trusts, and the presence or absence of external pressures, for example financial or regulatory challenges. Although some contextual factors can be modified by Trusts (for example, organisational cultures and leadership) many can not (for example, government policy) and, therefore, need to be taken into account by those implementing improvement programmes[24]

GIRFT is one of the largest improvement initiatives in the NHS. Following its introduction in orthopaedics, it was rolled out at substantial cost in over 40 other clinical areas, without independent evaluation. We have previously noted that changes to orthopaedic practice over the past 10 years are likely attributable to both GIRFT and other concurrent initiatives.[9] Our findings here help explain why the additional impact of GIRFT visits to individual Trusts was mixed: although all case sites had made improvements to care, very few were directly attributed to GIRFT. A range of factors, operating at different levels, also influenced sites' ability to implement GIRFT recommendations. As well as early engagement with rigorous evaluation design, those delivering future improvement programmes should consider how their intervention sits in relation to the three levels of context (macro, meso and micro) and the dynamic interactions that may occur between them. This is especially so given the advent of Integrated Care Systems (ICSs) which, until they are embedded, might create more complexity, but which have the potential to improve information flow and collaboration as these multi-level contexts become more integrated and dynamic relationships between the contextual levels change.

For peer review only - http://bmjopen.bmj.com/site/about/guidelines.xhtml

DECLARATIONS

Acknowledgements: We thank Patricia Hallam for administrative support for the study. We thank all the other members of the research team who contributed to other parts of this mixed methods evaluation. We thank the public members of the NIHR CLAHRC/ARC North Thames Research Advisory Panel who commented on all aspects of the study and the professional stakeholders who gave their time to take part in interviews and provide relevant documentation. We thank the GIRFT programme team for providing lists of sites from which to recruit case sites anonymously.

Contributors: HB was principal investigator. HB and RR initiated the research. HB, NJF and RR designed the evaluation. SJ and JL were responsible for qualitative data collection; FA, JL, SJ, RM, NJF and HB analysed the qualitative data. FA, JL, SJ, RR, NJF and HB drafted the manuscript. All authors contributed to reviewing and substantive revision. All authors approved the final version. All authors had full access to all the data in the study and accept responsibility to submit for publication. HB will act as guarantor.

Funding: This reports independent research funded by the National Institute for Health Research (NIHR) Collaboration for Leadership in Applied Health Research and Care (CLAHRC), North Thames. The NIHR Applied Research Collaboration (ARC) North Thames provided a costed extension (award/grant number is not applicable).

Disclaimer: This report is independent research funded by the National Institute for Health Research ARC North Thames. The views expressed in this publication are those of the authors and not necessarily those of the National Institute for Health Research or the Department of Health and Social Care.

Competing interests: All authors have completed the ICMJE uniform disclosure form at www.icmje.org/coi disclosure.pdf, and declare: FA, JL, SJ, HB, RM, RR, HB are funded in full or in part by the National Institute for Health Research ARC North Thames. RR and NJF are NIHR Senior Investigators; no financial relationships with any organisations that might have an interest in the submitted work in the previous 3 years; no other relationships or activities that could appear to have influenced the submitted work.

Ethics approval and consent to participate: This study involved human participants and was reviewed and approved by NHS North West - Liverpool East Research Ethics Committee (16/NW/0654). Before taking part, all participants gave informed consent to participate in the study and for be analysed and reported anonymously.

Provenance and peer review: Not commissioned; externally peer reviewed.

Data availability statement: Due to the professional sensitivity of these data, qualitative data are not publicly available. Informed consent did not extend to use of these data outside the study.

Consent for publication – Not applicable

For peer review only

Availability of data and materials – Consent was not given by participants to share their anonymised transcripts.

ORCID iDs:

Fiona Aspinal http://orcid.org/0000-0003-3170-7570

Jean Ledger http://orcid.org/0000-0003-2523-7971

Sarah Jasim http://orcid.org/0000-0003-3940-6350

Raj Mehta http://orcid.org/0000-0002-4003-530X

Rosalind Raine http://orcid.org/0000-0003-0904-749X

Naomi J Fulop http://orcid.org/0000-0001-5306-6140

Helen Barratt http://orcid.org/0000-0002-1387-137X

For peer review only

REFERENCE LIST

1. Culliford D, Maskell J, Judge A, et al. Future projections of total hip and knee arthroplasty in the UK: results from the UK Clinical Practice Research Datalink. *Osteoarthritis and Cartilage* 2015;23(4):594-600. doi: 10.1016/j.joca.2014.12.022
2. OECD. Hip and knee replacement: Health at a Glance 2019. OECD Indicators. Paris: OECD Publishing 2019.
3. Gunsche JL, Pilz V, Hanstein T, et al. The variation of arthroplasty procedures in the OECD Countries: analysis of possible influencing factors by linear regression. *Orthopedic Reviews* 2020;12(3):118-23. doi: 10.4081/or.2020.8526
4. Abdelaal MS, Restrepo C, Sharkey PF. Global Perspectives on Arthroplasty of Hip and Knee Joints. *Orthopedic Clinics of North America* 2020;51(2):169-+. doi: 10.1016/j.ocl.2019.11.003
5. Carr A, Smith JA, Camaradou J, et al. Growing backlog of planned surgery due to covid-19. *BMJ* 2021;372:n339.
6. Briggs T. Getting it right first time. *Improving the quality of orthopaedic care within the National Health Service in England* 2012
7. Briggs T. A national review of adult elective orthopaedic services in England. *Getting it right first time* 2015;7
8. GIRFT. Getting it Right first Time 2022 [Available from: https://www.gettingitrightfirsttime.co.uk/girft-methodology/. accessed 16/04/2022 2022.
9. Barratt H, Hutchings A, Pizzo E, et al. Mixed methods evaluation of the Getting it Right First Time programme in elective orthopaedic surgery in England: an analysis from the National Joint Registry and Hospital Episode Statistics. *BMJ open* 2022;12(6):e058316.
10. Robert G, Fulop N. The role of context in successful improvement. *Perspectives on context A selection of essays considering the role of context in successful quality improvement London: Health Foundation* 2014;31
11. Horton T, Illingworth J, Warburton W. The spread challenge. *UK: Health Foundation* 2018
12. Dixon-Woods M, McNicol S, Martin G. Ten challenges in improving quality in healthcare: lessons from the Health Foundation's programme evaluations and relevant literature. *Bmj Quality & Safety* 2012;21(10):876-84. doi: 10.1136/bmjqs-2011-000760
13. Currie G, Spyridonidis D. Sharing leadership for diffusion of innovation in professionalized settings. *Human Relations* 2019;72(7):1209-33. doi: 10.1177/0018726718796175
14. Fulop NJ, Ramsay AIG. How organisations contribute to improving the quality of healthcare (vol 365, l1773, 2019). *Bmj-British Medical Journal* 2019;366 doi: 10.1136/bmj.l4496
15. Burgoyne J, Williams S, Walmsley J. Evaluation of the Shared Leadership for Change Award Scheme. *London: The Health Foundation* 2009
16. Barratt H, Turner S, Hutchings A, et al. Mixed methods evaluation of the Getting it Right First Time programme - improvements to NHS orthopaedic care in England: study protocol. *BMC Health Services Research* 2017;17(1):71.
17. Yin RK. Case study research: Design and methods. Thousand Oaks. *Sage Young, LC and Wilkinson, IR (1989) The role of trust and co-operation in marketing channels: a preliminary study European Journal of Marketing* 2003;23(2):109-22.
18. Yin RK. Validity and generalization in future case study evaluations. *Evaluation* 2013;19(3):321-32.
19. Bowling A. Research Methods in Health. Open University Press. *Maidenhead, England* 2002
20. Braun V, Clarke V. Contextualism. thematic analysis a practical guide: London: Sage Publications, 2022.
21. Lingard L. Beyond the default colon: Effective use of quotes in qualitative research. *Perspectives on Medical Education* 2019;8(6):360-64. doi: 10.1007/s40037-019-00550-7
22. British Orthopaedic Association. BOA Professional Guidance to Implement Getting it Right First Time in England. London: BOA, 2016.

For peer review only - http://bmjopen.bmj.com/site/about/guidelines.xhtml

23. GIRFT. Reflecting on success and reinforcing improvement: a follow-up on the GIRFT national specialty report on orthopaedics. London: GIRFT, 2020.
24. Fulop N, Robert G. Context for successful quality improvement: Health Foundation 2015.

For peer review only

For peer review only - http://bmjopen.bmj.com/site/about/guidelines.xhtml

Topic guide T1 and T2 – format amended for publication

GIRFT: T1 Final topic guide for provider interviews

Background checks / intro:

- Check respondent aware of aims of the study (e.g., reviewed information sheet)
- Seek permission to record.
- Confirm role in the Trust.

Priority questions are highlighted in yellow

A) Experience of GIRFT programme and site visits

1. Please describe your current understanding of the GIRFT programme in orthopaedics.
   - What do you think are the main problems or practices that the GIRFT team want to address? (N.B. establish knowledge of GIRFT priorities ***unprompted***)

**Intervention priority**	**Interviewer ticks all that are mentioned**
Procurement costs / Trust expenditure:<br>- Implants selection/supply (e.g. cemented / uncemented, prosthesis brand)<br>- Equipment costs (e.g. theatre loan kits)	
NHS Litigation costs	
Oxford hip / knee scores (OHS/OKS) which are indicative of primary total hip / knee replacements (PROMS adjusted health gains)	
5-year revisison rates	
Surgeon activity levels (i.e. % of surgeons undertaking less than 5 procedures per year)	
Readmission rates (i.e. for post surgical complications)	
Infection rates	
Length of Stay (primary total hip / knee replacements)	
Knee replacements following an arthroscopy after 1 year	
Other	[Enter details]

2. When did you first hear about GIRFT? [PROMPT:]
   - How was it introduced to you within this hospital? By whom?

3. Have you been involved in a GIRFT visit to this hospital? [**If yes – continue to Q.4**]:

4. Please describe your experience of this ***first visit*** by the GIRFT team ? [PROMPTS]:
   - When did this take place? [N.B. cross-check dates]
   - How long was the meeting?

For peer review only - http://bmjopen.bmj.com/site/about/guidelines.xhtml

Topic guide T1 and T2 – format amended for publication

- Did you prepare for the meeting in advance? If so, what did you do? How much time did you spend on this?
    i. Did you review the data pack sent in advance of the meeting? [N.B. cross-check dates]
- What was your impression of the data presented in the meeting? Did any aspects surprise you?
- Do you recall the main issues raised and discussed during that meeting?
- Was there anything pointed out by the GIRFT team that you found particularly helpful? And anything you found particularly challenging?
- What were your expectations before the visit? Did the meeting meet your expectations?
- Had you experienced anything like this before? If so, what was different about it?

5. What steps were taken following the *first* visit? [PROMPTS:]
    - Can you give us examples of any conversations that took place between senior managers and clinicians following the first GIRFT visit?
    - Who was involved in any decisions made after the visit?
    - What actions were agreed to take forward? (If any) [Fill out the table below]

Description of action taken	People involved	Time taken / duration	Costs involved / resources required for implementation (e.g. staff time, materials, capital)

6. Have you been involved in a GIRFT *re-visit* at this Trust? [PROMPTS:]
    - When did this take place? [N.B. cross-check dates]
    - How long was the meeting?
    - Did you prepare for the meeting in advance? If so, what did you do? How much time did you spend on this?
        i. Did you review the data sent in advance of the meeting? [N.B. cross-check dates]
    - Do you recall the main issues raised / discussed during this meeting?
    - Did it differ from the first meeting (if you were present)?
        - What steps were taken following this meeting? Who was involved in these decisions?

For peer review only - http://bmjopen.bmj.com/site/about/guidelines.xhtml
For peer review only

Topic guide T1 and T2 – format amended for publication

Description of action taken	People involved	Time taken / duration	Costs involved / resources required for implementation (e.g. staff time, materials, capital)

B) Other local quality improvement initiatives:

7. Are you involved in any national or local quality improvement initiatives focused on orthopaedic services? [PROMPTS – if yes:]
   - What are the aims of this initiative? [i.e. only non-GIRFT programmes]
   - When did the initiative begin?
   - What has been your level of involvement?
   - How is the initiative attempting to change orthopaedic services or practice?

C) The Model Hospital

8. Have you heard of the Model Hospital Orthopaedic dashboard? [PROMPTS:]
   - [If yes] What do you know about it?
   - Do you think it will help to makes changes in this Trust? How?

D) Changes processes and attribution to the GIRFT programme (i.e. changes to practice or processes, clinical and financial outcomes)

9. In your opinion, has GIRFT made a difference within this Trust? If so, please can you talk us through some examples? [PROMPTS]:
   - Have you made any changes to organisational practices around: 1) procurement; 2) litigation; 3) financial management; 4) clinical coding; 5) service delivery; 6) patient care?
   - What are the timescales for delivering these specific changes to practice? When did work begin? (i.e. explore short-term vs. medium-term vs. long-term)
   - Do you perceive any changes in how resources or finances are allocated in this Trust because of GIRFT interventions?
   - Have the GIRFT team helped you to deliver any of these changes?
   - Which changes in this hospital can be attributed to GIRFT in your opinion, and why?

For peer review only - http://bmjopen.bmj.com/site/about/guidelines.xhtml

Topic guide T1 and T2 – format amended for publication

- Can you tell us about any unintended consequences brought about as a result of GIRFT?
- Do you measure progress or success in relation to GIRFT within the Trust? [PROMPTS]:
- Do you measure cost savings arising from the GIRFT intervention?

E) Local context

10. Is there anything we should be aware of about the clinical coding system used at this Trust?
11. Is there anything that makes it difficult to implement the GIRFT recommendations in this site?
12. Is there anything - or anyone - that has really helped to support implementation of the GIRFT recommendations?

Wrap up & close

F) What improvements could be made to the GIRFT programme in future?

For peer review only

Topic guide T1 and T2 – format amended for publication

GIRFT: T2 Topic Guide for Follow-up Provider Interviews

Background checks / intro:

- Check respondent aware of aims of the study (e.g., reviewed information sheet)
- Seek permission to record.
- Confirm role in the Trust (especially if changed since T1 interview)
- [If possible, use presentation given at the GIRFT re-visit as a discussion prompt]

A) Implementation of GIRFT recommendations since last interview (T1)

1. Has this unit implemented any GIRFT recommendations in trauma and orthopaedics since [DATE OF T1 INTERVIEW]? Prompt list:

For peer review only

Changes in:	Tick those that apply
**Finance and procurement:**	
• Procurement processes (e.g., new contracts, rationalisation of implants)	
• Use of theatre loan kit (e.g., reduction in expenditure)	
**Infection control, bed management and theatre efficiency:**	
• Ring fenced beds	
• New infection control policies	
• Data collection on infection rates	
• Length of Stay (e.g., new protocols)	
• Number of cases performed per day (e.g., minimum of 4 THR/TKR)	
**Rehabilitiation and physiotherapy:**	
• Appointment of weekend cover	
• New patient education materials	
**Surgeon-level clinical practice:**	
• Surgeon minimum volumes (e.g. recommendations for thresholds for doing procedures)	
• Cemented and / or uncemented implants in patients aged over 65/70	
• Implant selection (e.g. bearings, type of supplier)	
**Team-or network-level practice:**	
• Multi-disciplinary teams (e.g. for review of revisions within unit or regional network)	
• Creation of committees / meetings (e.g. GIRFT implementation team)	
• Review of performance or outcomes data to inform practice (e.g. NJR, litigation cases)	
**Workforce and service re-configuration:**	
• Appointment of orthogeritrician/physiotherapists/shift in number of consultants (especially if low volume)	
• ‘Hot and cold’ sites	

For peer review only - http://bmjopen.bmj.com/site/about/guidelines.xhtml

Topic guide T1 and T2 – format amended for publication

2. When did work [on X items listed above, if applicable] begin? What has facilitated this? [Explore *how* the work has been facilitated].

3. If you have not been able to implement any specific GIRFT recommendations, or decided not to, please explain why.

4. Have any GIRFT recommendations been particularly difficult to implement locally? If so, what factors have made implementation difficult? Why?

   Prompts:

• Winter bed pressures	Tick any that apply
• Workforce issues (e.g. lack of theatre staff/physiotherapists, recruitment and retention)	
• Lack of funds (e.g. for ring fenced beds)	
• Referral to treatment (RTT) targets	
• New tariff	
• Other	

5. Have there been any controversies locally around particular GIRFT recommendations in orthopaedics? (e.g., uncemented vs. cemented implant use) What impact have these had (if any)?

6. How are you measuring progress in relation to GIRFT within the Trust?

   PROMPTS:

   - Cost savings (e.g., theatre loan kit reduction)
   - NJR compliance
   - Less lower volume procedures
   - Lower infection rates

7. Can you tell us about any knock-on effects brought about as a result of GIRFT so far? (e.g., impact on trauma work in orthopaedics)

B) GIRFT orthopaedic re-visits 2018

1. To confirm, when did this site have a GIRFT orthopaedic re-visit with the national lead?

For peer review only - http://bmjopen.bmj.com/site/about/guidelines.xhtml

Topic guide T1 and T2 – format amended for publication

2. Who was invited to attend? Who was unable to attend? Did anyone else want to attend?

3. How did you prepare for the meeting? [Prompt]:
    - Preparation of presentation using GIRFT in advance of the visit

4. Did the most recent meeting differ from the first visit (if you were present)? If so, how?

5. Has anything happened since this re-visit? [Prompt]:
    - Are there GIRFT recommendations you are planning to implement in the next 6 months?

C) Other local quality improvement initiatives and research

1. Are you involved in any other quality improvement initiatives or research studies focused on orthopaedic services that relate to GIRFT orthopaedics?

D) The Model Hospital

1. Are you currently using the Model Hospital Orthopaedic dashboard? [PROMPTS:]
    - [If yes] What is your understanding?
    - Have you used it to make any changes in your Trust?
    - Do you think it will help to makes changes in this Trust? How?

E) GIRFT national and regional engagement

1. Have you liaised with any of the GIRFT implementation hubs / managers? [Prompt]:
    - What is your understanding of their role?
    - How this could this support implementation of GIRFT recommendations in your view?

F) Wrap up & close:

1. Are you aware of any changes in the national GIRFT programme since we last spoke?
2. PROMPT for awareness of evolution of national programme in relation to orthopaedics
3. Do you have any recommendations for improvements which could be made to the GIRFT programme?
4. Do you have any further comments?

Thanks and close

For peer review only - http://bmjopen.bmj.com/site/about/guidelines.xhtml

Standards for Reporting Qualitative Research (SRQR)*

http://www.equator-network.org/reporting-guidelines/srqr/

Title and abstract

	Page/line no(s).
**Title** - Concise description of the nature and topic of the study Identifying the study as qualitative or indicating the approach (e.g., ethnography, grounded theory) or data collection methods (e.g., interview, focus group) is recommended	1
**Abstract** - Summary of key elements of the study using the abstract format of the intended publication; typically includes background, purpose, methods, results, and conclusions	2

Introduction

**Problem formulation** - Description and significance of the problem/phenomenon studied; review of relevant theory and empirical work; problem statement	4
**Purpose or research question** - Purpose of the study and specific objectives or questions	5

Methods

**Qualitative approach and research paradigm** - Qualitative approach (e.g., ethnography, grounded theory, case study, phenomenology, narrative research) and guiding theory if appropriate; identifying the research paradigm (e.g., postpositivist, constructivist/ interpretivist) is also recommended; rationale**	5
**Researcher characteristics and reflexivity** - Researchers' characteristics that may influence the research, including personal attributes, qualifications/experience, relationship with participants, assumptions, and/or presuppositions; potential or actual interaction between researchers' characteristics and the research questions, approach, methods, results, and/or transferability	
**Context** - Setting/site and salient contextual factors; rationale**	5
**Sampling strategy** - How and why research participants, documents, or events were selected; criteria for deciding when no further sampling was necessary (e.g., sampling saturation); rationale**	5 & 6
**Ethical issues pertaining to human subjects** - Documentation of approval by an appropriate ethics review board and participant consent, or explanation for lack thereof; other confidentiality and data security issues	19
**Data collection methods** - Types of data collected; details of data collection procedures including (as appropriate) start and stop dates of data collection and analysis, iterative process, triangulation of sources/methods, and modification of procedures in response to evolving study findings; rationale**	6

For peer review only

For peer review only - http://bmjopen.bmj.com/site/about/guidelines.xhtml

**Data collection instruments and technologies** - Description of instruments (e.g., interview guides, questionnaires) and devices (e.g., audio recorders) used for data collection; if/how the instrument(s) changed over the course of the study	6
**Units of study** - Number and relevant characteristics of participants, documents, or events included in the study; level of participation (could be reported in results)	7 & 8
**Data processing** - Methods for processing data prior to and during analysis, including transcription, data entry, data management and security, verification of data integrity, data coding, and anonymization/de-identification of excerpts	6
**Data analysis** - Process by which inferences, themes, etc., were identified and developed, including the researchers involved in data analysis; usually references a specific paradigm or approach; rationale**	6 & 7
**Techniques to enhance trustworthiness** - Techniques to enhance trustworthiness and credibility of data analysis (e.g., member checking, audit trail, triangulation); rationale**	5, 6 & 19

Results/findings

**Synthesis and interpretation** - Main findings (e.g., interpretations, inferences, and themes); might include development of a theory or model, or integration with prior research or theory	8-14
**Links to empirical data** - Evidence (e.g., quotes, field notes, text excerpts, photographs) to substantiate analytic findings	8-14

Discussion

**Integration with prior work, implications, transferability, and contribution(s) to the field** - Short summary of main findings; explanation of how findings and conclusions connect to, support, elaborate on, or challenge conclusions of earlier scholarship; discussion of scope of application/generalizability; identification of unique contribution(s) to scholarship in a discipline or field	14-15
**Limitations** - Trustworthiness and limitations of findings	3 & 15

Other

**Conflicts of interest** - Potential sources of influence or perceived influence on study conduct and conclusions; how these were managed	19
**Funding** - Sources of funding and other support; role of funders in data collection, interpretation, and reporting	19

*The authors created the SRQR by searching the literature to identify guidelines, reporting standards, and critical appraisal criteria for qualitative research; reviewing the reference lists of retrieved sources; and contacting experts to gain feedback. The SRQR aims to improve the transparency of all aspects of qualitative research by providing clear standards for reporting qualitative research.

For peer review only

For peer review only - http://bmjopen.bmj.com/site/about/guidelines.xhtml

**The rationale should briefly discuss the justification for choosing that theory, approach, method, or technique rather than other options available, the assumptions and limitations implicit in those choices, and how those choices influence study conclusions and transferability. As appropriate, the rationale for several items might be discussed together.

Reference:

O'Brien BC, Harris IB, Beckman TJ, Reed DA, Cook DA. **Standards for reporting qualitative research: a synthesis of recommendations.** *Academic Medicine*, Vol. 89, No. 9 / Sept 2014 DOI: 10.1097/ACM.0000000000000388

For peer review only
